# Long‐term fire and vegetation change in northwestern Amazonia

**DOI:** 10.1111/btp.13175

**Published:** 2022-12-02

**Authors:** Britte M. Heijink, Quinten A. Mattijs, Renato Valencia, Annemarie L. Philip, Dolores R. Piperno, Crystal N. H. McMichael

**Affiliations:** ^1^ Department of Ecosystem and Landscape Dynamics, Institute for Biodiversity and Ecosystem Dynamics University of Amsterdam Amsterdam The Netherlands; ^2^ Escuela de Ciencias Biológicas Pontificia Universidad Católica del Ecuador Quito Ecuador; ^3^ Department of Anthropology Smithsonian National Museum of Natural History Washington District of Columbia USA; ^4^ Smithsonian Tropical Research Institute Balboa Panama

**Keywords:** charcoal, ecological legacies, forest plots, hyperdominants, past human disturbance, past vegetation change, phytoliths, tropical forest

## Abstract

Amazonian forest plots are used to quantify biodiversity and carbon sequestration, and provide the foundation for much of what is known about tropical ecology. Many plots are assumed to be undisturbed, but recent work suggests that past fire, forest openings, and cultivation created vegetation changes that have persisted for decades to centuries (ecological legacies). The Yasuní Forest Dynamics plot is one of the most biodiverse places on earth, yet its human history remains unknown. Here, we use charcoal and phytolith analysis to investigate the fire and vegetation history of the Yasuní forest plot, and compare results with nearby forest plots in Colombia (Amacayacu) and Peru (Medio Putumayo‐Algodón [MPA]) to explore the spatial variability of past disturbances and ecological legacies in northwestern Amazonia. Three ^14^C dated charcoal fragments provided evidence for a modern (1956 CE) and a past fire event ca. 750 years ago at Yasuní, compared with fire ages of 1000–1600 years ago documented at Amacayacu and MPA. Small‐scale disturbances and localized canopy openings also occurred in the Yasuní plot. Phytolith assemblages from Yasuní and Amacayacu showed more variability in past vegetation change than MPA. Low‐intensity, non‐continuous disturbances occurred at all three plots in the past, and our results highlight the variability of past human activities both in space and time in northwestern Amazonia. Our data also suggest that post‐Columbian human disturbances from the Rubber Boom (AD 1850–1920) and subsequent oil exploration have likely left stronger ecological legacies than those left by pre‐Columbian peoples in our studied regions.

## Graphical abstract

Forest plots in Amazonia are often assumed to be undisturbed, but recent work suggests that past people can affect modern vegetation composition in the form of ecological legacies. We investigated the past fire and vegetation history of the Yasuní Forest Dynamics plot and compared that to other forest plots in northwestern Amazonia. Our data suggest that post‐Columbian human disturbances from the Rubber Boom (AD 1850‐1920) and subsequent oil exploration have likely left stronger ecological legacies than those left by pre‐Columbian peoples in northwestern Amazonia.

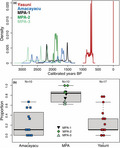

## 1 INTRODUCTION

**FIGURE 1:**
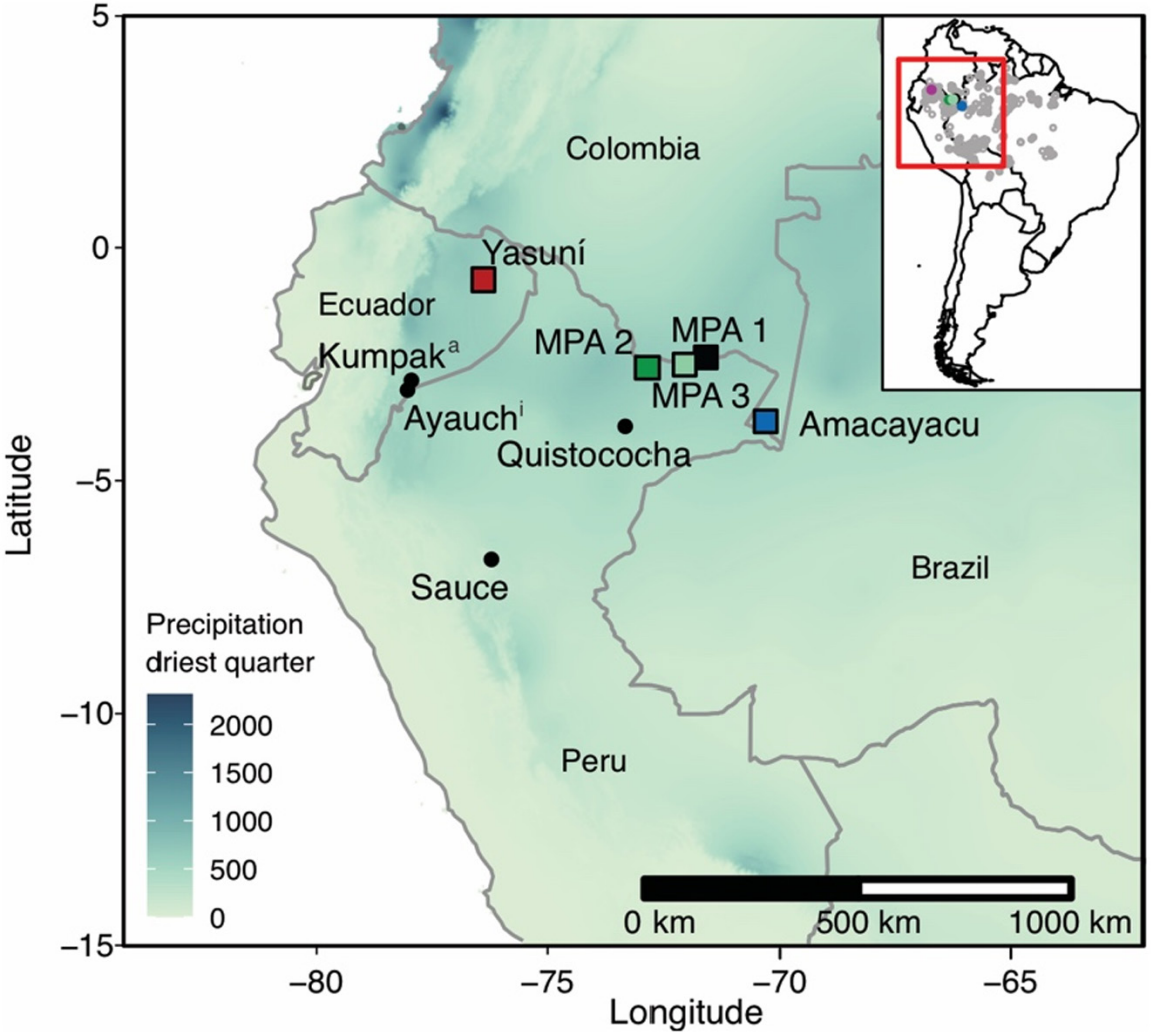
Locations of Yasuní Forest dynamics plot, Amacayacu Forest dynamics plot, and Medio Putumayo‐Algodón (MPA) rapid inventory plots and other sites mentioned in the text. Background is the precipitation of the driest quarter (driest 3 months per year) (Hijmans et al., 2005). The inset shows the distribution of Amazonian forest plots in gray.

Forest plots in tropical forests such as the Amazon are vital to understanding patterns of biodiversity (Myers et al., 2000; Slik et al., 2015), the relative abundances of species (Gentry, 1988; Pitman et al., 2001; ter Steege et al., 2013), and ecosystem functions such as carbon sequestration (Fauset et al., 2015; Pan et al., 2011; Phillips et al., 1998). Over 1200 forest plots exist within the 6 million km^2^ of Amazonian forests, and more than 321 plots are censused repeatedly to investigate carbon dynamics (Figure 1) (Brienen et al., 2015; Draper et al., 2021; ter Steege et al., 2013). Data from these plots suggest that over 15,000 tree species occur in Amazonia but only 1.4% of those species account for 50% of all tree individuals (>10 cm DBH) (ter Steege et al., 2013, 2020). These “hyperdominant” trees have high population sizes, and thus also play a large role in carbon storage and forest productivity (Fauset et al., 2015). The driving factors behind these patterns of Amazonian hyperdominance remain unclear, but habitat heterogeneity, other ecological and evolutionary factors that promote competitive influences, and past human influences have been proposed as possible explanations (Levis et al., 2017; ter Steege et al., 2013).

People have occupied Amazonia and used fire for more than 13,000 years (Gosling et al., 2021; Roosevelt, 2013). Fire was used to clear land and add nutrients to soils, and charcoal produced from fire is a prime component of anthrosols (human‐created soil types) called *terras pretas*, Amazonian Black Earth, or Amazonian Dark Earth (e.g., Glaser & Woods, 2004; Lehman et al., 2003). However, fire usually does not ignite and spread without human propagation in Amazonian rainforests, and most plants are not evolutionarily adapted to fires (Barlow & Peres, 2008; Uhl & Kauffman, 1990). Repeated burns can lead to a complete species composition turnover in these tropical rainforest trees (Barlow & Peres, 2008). Forest recovery of biomass can take over 100 years (Poorter et al., 2016), and species composition likely takes even longer (Chazdon, 2003; Loughlin et al., 2018).

Besides fire, past people also directly impacted past vegetation composition in Amazonia by domestication and cultivation, enriching abundances of useful species, and depleting abundances of other useful species (e.g., Balée, 1988, 1989; Clement et al., 2010, 2015). The effects of past human activities on vegetation composition and carbon dynamics can last hundreds of years due to the longevity of tropical trees and slow recovery processes (Hartshorn, 1978; Poorter et al., 2016; Rozendaal et al., 2019). The long‐term effect of these past human activities on modern ecosystems is typically called an “ecological legacy” (Franklin et al., 2000; Turner, 2010). Recognizing and accounting for these ecological legacies are vital to understanding the current patterns and processes documented in modern Amazonian forests. (Levis et al., 2017; McMichael, 2021).

Palms are one of the most widely used plant groups of both previous and current inhabitants of Amazonia (Bernal et al., 2011; Clement et al., 2015). Palms with edible fruits, such as *Bactris gasipaes* (a domesticated species) and *Mauritia flexuosa*, are believed to have been enriched in the pre‐Columbian era and have persisted in high abundances into modern times (Clement, 1988; Clement et al., 2015; Rull & Montoya, 2014). In turn, other palms such as *Wettinia maynensis* or *Iriartea deltoidea*, whose solitary stem is usually harvested, may have been depleted by past humans (Åkesson et al., 2021; Bush & McMichael, 2016). Interestingly, *I. deltoidea* has shown an ability to rebound to higher abundances after site abandonment. These data collectively suggest a relationship between past human activity and modern palm abundances. However, increases in overall precipitation over the last 4000 years and other ecological and evolutionary factors may have also played a role in structuring palm abundances (Bush & McMichael, 2016; Heijink et al., 2020).

Past human activities have only been reconstructed in 5 of the ca. 1240 forest plots across Amazonia, typically by analyzing charcoal and phytoliths extracted from soil cores collected within the plot (Heijink et al., 2020; McMichael et al., 2012, 2015; Piperno et al., 2021; Piperno & Becker, 1996). Phytoliths are silica microfossils produced by many Neotropical plants that preserve well in soils and sediments and can be used to detect past cultivation, canopy openings, and changes in tree taxa (e.g., Dickau et al., 2013; Piperno, 2006; Piperno & McMichael, 2020; Watling et al., 2016). Palms are abundant phytolith producers, and recent work has shown that phytoliths can be used identify palms at the genus level, particularly when the local species pool is known (Huisman et al., 2018; Morcote‐Ríos et al., 2016; Piperno et al., 2021; Piperno & McMichael, 2020; Witteveen et al., 2022).

Here, we reconstruct the spatial and temporal patterns of past fires and vegetation change within one of the oldest and most heavily studied forest plots within northwestern Amazonia (Yasuní, Ecuador) (e.g., Bass et al., 2010; John et al., 2007; Pitman et al., 2001, 2013). We determine the ages of the most recent fire events and patterns of overall vegetation change, and compare our results with recently published charcoal and phytolith data from nearby forest plots in the Colombian and Peruvian Amazon (Heijink et al., 2020; Piperno et al., 2021) to investigate the spatial heterogeneity of past disturbances and ecological legacies in northwestern Amazonia.

## 2 METHODS

### 2.1 Site description and phytolith and charcoal sampling

The Yasuní National Park and Biosphere Reserve (Figure 1) in the Ecuadorian Amazon rainforest consists of *terra firme* and seasonally inundated forests (Valencia, Condit, et al., 2004; Valencia, Foster, et al., 2004). Yasuní has an aseasonal climate, receives ±3000 mm of rainfall per year with no dry season (monthly rains always >100 mm), maintains year‐round humidity between 80 and 94%, and the temperature ranges between 24 and 27 °C (Valencia, Condit, et al., 2004). The soils are geologically young and consist mostly of fluvial sediments eroded from the Andes (Valencia, Foster, et al., 2004). The Yasuní National Park is the ancestral land of the Waorani (Huaorani) but is now also inhabited by the Kichwaruna (Quichua) people (Finer et al., 2009).

The Yasuní Forest Dynamics Plot (hereafter Yasuní) (−0.68, −76.40) is 50 hectares in size (1000 m x 500 m) and was established within the Yasuní National Park in 1995 by the Pontifical Catholic University of Ecuador (Valencia, Condit, et al., 2004). It is part of ForestGEO, a global network of large scale forest plots (https://forestgeo.si.edu/). Elevations within the plot range between 230 and 270 masl (meters above sea level) (Valencia, Condit, et al., 2004). The average slope in the plot is 13% and two ridges surround a wetter valley near the center of the plot (Queenborough et al., 2007; Valencia, Condit, et al., 2004). In the western part of the plot, all stems are censused at >1 cm DBH (diameter at breast height), and in the eastern part of the plot, all stems are censused >10 cm DBH (Valencia, Foster, et al., 2004). A small swamp is present in the eastern side of the plot. Over 150,000 individual trees have been recorded in the western half of the plot, comprising 1104 species (Valencia, Foster, et al., 2004). The forest plot is reported to contain primarily old‐growth forests except in the southwest corner (Valencia, Condit, et al., 2004), though ceramics with an estimated age of 500–1000 years were found near the northwestern corner of the plot (Netherly, 1997; Valencia, Condit, et al., 2004).

From within the Yasuní forest plot, 17 randomly located soil cores were collected in 10 cm depth intervals to a total depth of 80 cm (N = 9) in July 2018 (Figure S1a). We analyzed 17 soil cores for charcoal to reconstruct past fire history (N = 153), and 10 of those soil cores were analyzed for phytoliths to reconstruct past vegetation history (N = 90) (Figure S1a). Pinch samples of the surficial soils (4–6 per location), or top soil, were also collected within a 3‐meter radius of each soil core to obtain phytolith assemblages that represent modern vegetation (N = 10).

### 2.2 Laboratory analyses

Charcoal analysis was performed on each sample using 9.5 to 22 ml of initial soil volume. Each sample was boiled for 20 minutes at 150°C in 10% H_2_O_2_ and rinsed and sieved at 500 μm. Charcoal fragments were identified based on characteristics described in Scott (2010) using an Olympus SZH10 stereoscope. Charcoal particles were photographed with a Fuji camera, and the surface area of each fragment was calculated with Image J software (Rasband, 2005). The surface area of each charcoal fragment was converted to a volumetric measurement (cubic centimeters, cm^3^) by raising it to the power of 3/2 (Weng, 2005). We calculated the charcoal volume per cubic centimeter of soil by summing the volume of all charcoal fragments per sample and dividing by the original soil volume.

From each sample used in phytolith analysis (N = 90), we subsampled 1 cm^3^ of soil. We used HCl, H_2_O_2_, and KMnO_4_ to remove organics, carbonates, and humic acids from each sample. Phytoliths were separated by heavy liquid flotation from remaining soil material using bromoform (CHBR_3_, specific gravity 2.3 g/cm^3^). Phytoliths were mounted on microscope slides using Naphrax. We identified phytoliths using a Zeiss Axioscope.A1 at 1000x magnification (oil immersion). We counted a minimum of 200 arboreal and 300 total phytoliths (Aleman et al., 2013, 2014) for each sample. Extended scans for larger arboreal phytoliths (Piperno et al., 2019, 2021; Piperno & McMichael, 2020) were done at 200x without oil. Phytoliths were identified using published reference photographs (Huisman et al., 2018; Morcote‐Ríos et al., 2016; Piperno, 2006; Piperno & McMichael, 2020; Witteveen et al., 2022) and the phytolith reference collection of the University of Amsterdam.

### 2.3 Data analyses

Charcoal fragments larger than >1 mm^3^ were submitted to DirectAMS Laboratory (Seattle, Washington, USA) for radiocarbon dating (N = 3). The obtained ^14^C dates were calibrated, and cumulative probabilities for the timing of fire events were calculated using the “*Bchron”* package (Parnell, 2016) in R (R Development Core Team, 2013) using the IntCal20 calibration curve (Reimer et al., 2020). For each date, we calculated the 95% confidence interval and reported the minimum, median, and maximum calibrated age. We calibrated modern ages based on the percent modern carbon (pMC) using the “*IntCal”* package (Blaauw, 2021). Charcoal dated from other sites were recalibrated with the IntCal20 calibration curve for direct comparisons. To assess spatial patterns of past fires, we calculated total charcoal abundance per depth interval and per core. We also calculated the proportion of samples containing charcoal per core and per depth across all ten cores.

For each phytolith sample (N = 90), we calculated the relative percentage (%) of each morphotype encountered. For all main phytolith types, we calculated the trend of change (% surface sample ‐ % of basal sample) and magnitude of change (maximum % ‐ minimum %) to quantify vegetation changes within cores (McMichael et al., 2015). Trend of change represents the overall direction of change from bottom to top of the soil core, and magnitude of change represents the total amount of change within the core. These calculations were done on all phytolith types, with a particular focus on palms due to the high taxonomic resolution compared to arboreal phytoliths.

We compared our data from Yasuní with charcoal and phytolith data from the Amacayacu (Heijink et al., 2020) and Medio‐Putumayo Algodon (hereafter MPA) (Piperno et al., 2021) terra firme forest plots located in Colombia and Peru (Figure 1, Table 1). Amacayacu is a 25‐hectare forest plot where stems >1 cm diameter at breast height (DBH) have been measured twice since 2007. Amacayacu receives ca. 3200 mm of precipitation per year, with no dry season (Rudas Lleras et al., 2005). MPA is a region spanning hundreds of kilometers, where 1‐hectare surveys were performed in 2016 at three locations, Quebrada Bufeo (MPA‐1), Medio Algodon (MPA‐2), and Bajo Algodon (MPA‐3) (Pitman et al., 2004). Annual precipitation levels vary little across the MPA region and annual precipitation is 2893 mm/year.

**TABLE 1:** Overview of plots including size, number of cores taken, and past and modern disturbances. Precipitation data were extracted from Hijmans et al. (2005) from the period 1950–2000. The number of cores indicates the number of cores analyzed for phytoliths with the number of cores analyzed for charcoal between brackets (). Modern disturbances are defined as direct, anthropogenic disturbances likely present in the last 50 years.

Site	Size (h)	Precipitation driest quarter (mm)	No. cores	Evidence of pre‐Columbian pottery	Modern disturbances
Yasuní (Ecuador)	50	621	10 (17)	Yes	Large blow‐down of 96 trees (March 2002), abandoned helipad present
Amacayacu (Colombia)	25	507	10 (12)	No, nearest archeological site at 51 km (Morcote‐Rios et al., 2013)	‐
MPA (Peru)	3	635	10 (10)	Pottery found in the area of MPA‐1 4 km from the sampled area but it cannot be dated.	Hunting near MPA‐2. MPA‐3 is located near an abandoned logging concession

We used a species list of palms currently present in the Yasuní, Amacayacu, and MPA forest plots to aid in the interpretation of phytolith assemblages. We cross‐referenced all palm phytolith morphotypes found in our samples with the morphotypes produced by palms present in the species list. We classified all palm genera and species currently found in the forest plots as hyperdominant (ter Steege et al., 2013), useful to local indigenous peoples (Henderson et al., 1997; Macía, 2004; Macía et al., 2011), or neither.

The ages of past fires from Yasuni, Amacayacu, and MPA were compared using cumulative probability densities. Across the three sites we also compared: (i) charcoal abundances and (ii) the proportion of samples containing charcoal within a core using Kruskal–Wallis tests. Metrics for trend of change and magnitude of change were calculated for all phytolith types that were identified at Yasuní, Amacayacu, and MPA. We calculated z‐scores for each main phytolith type based on the percentages, and calculated the average z‐score per plot and depth interval to compare vegetation composition and vegetation change across plots and depths. We used the mean z‐scores per plot and depth interval to assess the distribution and magnitude of changes within and between soil cores. All data analyses were performed in R (R Development Core Team, 2013).

## 3 RESULTS

### 3.1 Charcoal data

**FIGURE 2:**
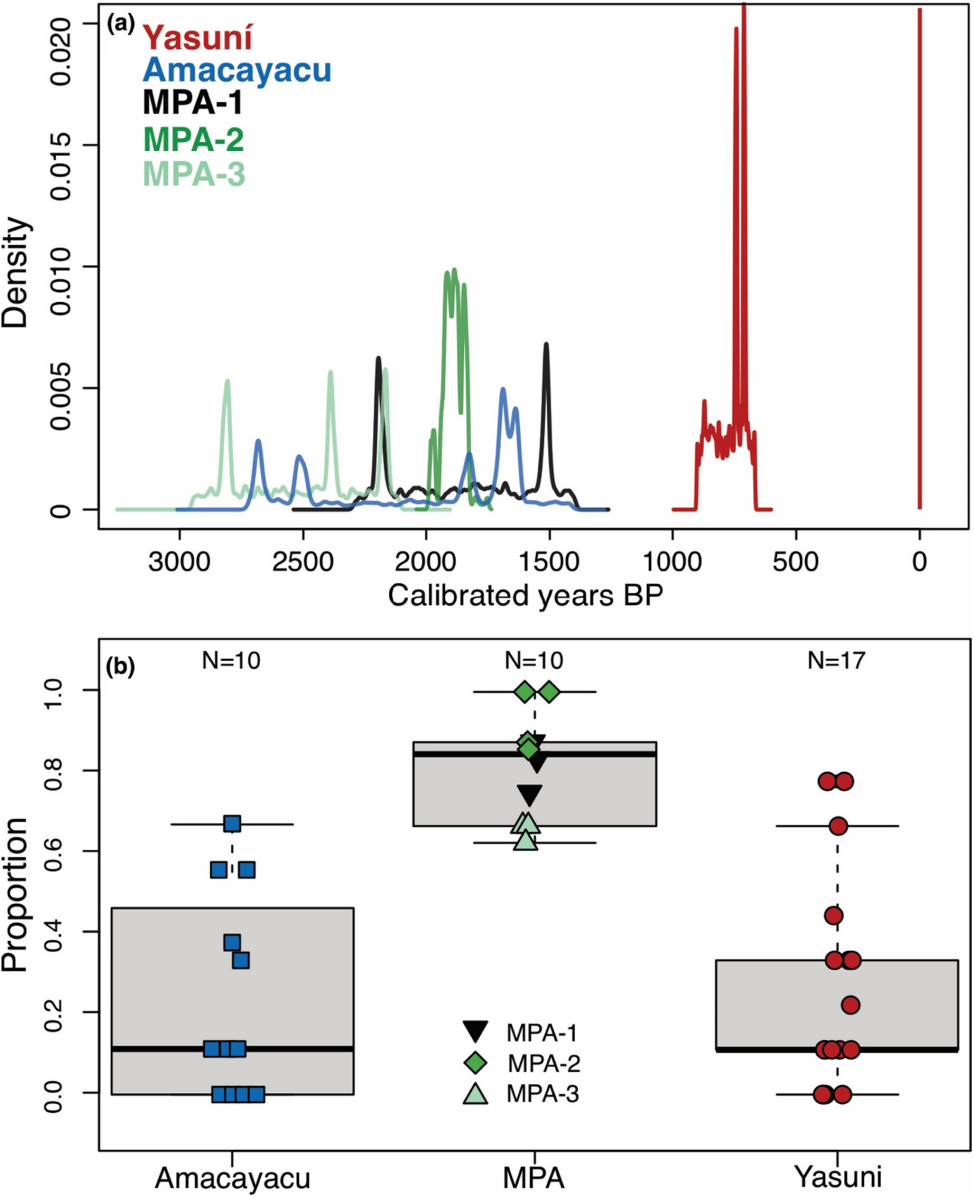
Spatial and temporal patterns of fire across three regions in northwestern Amazonia. (a) Cumulative probability densities of calibrated radiocarbon ages of Yasuní (red), Amacayacu (blue), and Medio Putumayo‐Algodón (darkgreen for MPA‐1, green for MPA‐2, light green for MPA‐3). The modern age at 1956 is indicated by the vertical red line. (b) Proportion of samples per core containing charcoal.

Only three charcoal fragments recovered from the 153 samples at Yasuní were large enough for ^14^C dating (Table S1, Figure 2). One fragment at Yasuní was indicated to be a modern fire event around 1956 CE (Common Era) that occurred in the southwest section of the plot near what is documented to be secondary forest (Table 1, Figure S1a) (Valencia, Condit, et al., 2004). The other two fragments were derived from fires that occurred in the northwestern portions of the Yasuní plot at ca. 750 cal yr BP (calibrated years Before Present) (1200 CE) (Table S1, Figure S1a).

**FIGURE 3:**
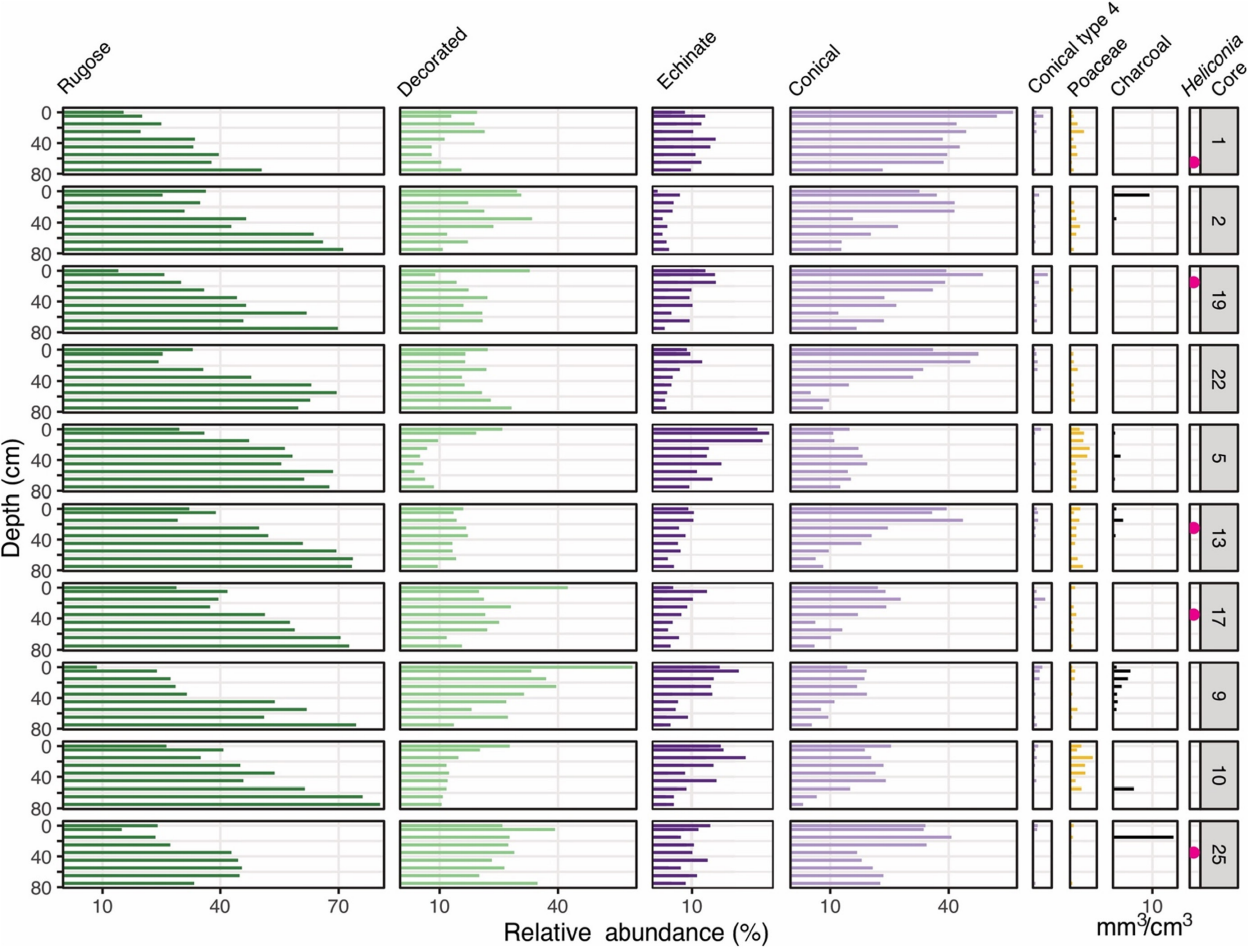
Stratigraphic diagram of phytolith and charcoal abundances found in soil cores in the Yasuní forest plot. Arboreal phytoliths are shown in green; palm phytoliths are shown in purple. Echinate shows the sum of all echinate phytolith types, including GE, GEE, GESP, reniform, and star palm. Conical shows the sum of all conical phytolith abundances, and Conical type 4 is included in that sum. Charcoal abundance is in mm^3^/cm^3^. The presence of *Heliconia* phytoliths is indicated with pink circles.

Four out of 17 cores at Yasuni lacked charcoal (Figures S2 and S1a), and only 25% of all samples (39 out of 153) contained charcoal (Table S2). Charcoal was most common from 0 to 20 cm depths and did not occur in the 70–80 cm depth interval (Figure 3).

### 3.2 Palm‐phytolith relationships

Only 2% of the species currently present in sizes >1 cm DBH in the Yasuní forest plot were palms (23 out of 1140), compared with 1.4% of species at Amacayacu and 1.6% at MPA. Of the 30 palm species and 16 genera found across the three sites (Table 2), 29 species are considered useful and 10 species are considered hyperdominant. *Geonoma aspidiifolia* was the only palm species occurring in Yasuní without a known use. Six palm species occurred at Amacayacu that were not found in Yasuní or MPA, and one palm species was found in MPA that did not occur in the other plots (Table 2).

**TABLE 2:** All palm species present in the Yasuní, Amacayacu, and MPA forest plots and the phytolith morphotypes they produce.

Tribe/subtribe	Species	Present at	Phytolith production
Lepidocaryeae/Mauritiinae	***Mauritia flexuosa***	Y, M	GE
Ceroxyloideae/Phytelepheae	*Phytelephas tenuicaulis*	Y	GE
Iriarteeae	***Iriartea deltoidea***	Y, A, M	Con 1
Iriarteeae	***Socratea exorrhiza***	Y, A, M	Con 1
Iriarteeae	*Wettinia maynensis*	Y	Con 2, Con 3
Chamaedoreeae	*Chamaedora pauciflora*	Y	Con 2, Con 3
Chamaedoreeae	***Chamaedora pinnatifrons***	Y	Con 2, Con 3
Cocoseae/Attaleinae	***Attalea butyracea***	A	GE, GEE
Cocoseae/Attaleinae	***Attalea maripa***	Y, M	GE, GEE
Cocoseae/Bactridinae	*Astrocaryum chambira*	Y, M	Con 1, Con 2, Con 3
Cocoseae/Bactridinae	***Astrocaryum ferrugineum***	A	Conical
Cocoseae/Bactridinae	***Astrocaryum murumuru***	M	Conical
Cocoseae/Bactridinae	*Astrocaryum urostachys*	Y	Con 1, Con 2, Con 3
Cocoseae/Bactridinae	*Aiphanes ulei*	Y	Con 2, Con 3
Cocoseae/Bactridinae	*Bactris corosilla*	Y	Con 1, Con 2, Con 3
Cocoseae/Bactridinae	*Bactris concinna*	A	Conical
Cocoseae/Bactridinae	*Bactris maraja*	Y, A	Con 1, Con 2, Con 3
Cocoseae/Bactridinae	*Bactris simplicifrons*	Y, A	Con 1, Con 2, Con 3
Cocoseae/Bactridinae	*Desmoncus giganteus*	A	Conical
Euterpeae	*Hyospathe elegans*	Y, A	GE, GEE, Reni
Euterpeae	***Euterpe precatoria***	Y, A, M	GE, GEE, GESP, LGG
Euterpeae	*Prestoea schultzeana*	Y	GEE, GESP, Reni
Euterpeae	***Oenocarpus bataua***	Y, A	GE, GEE, GESP
Euterpeae	*Oenocarpus mapora*	Y	GE, GEE, GESP
Euterpeae	*Oenocarpus minor*	A	GE, GEE, GESP, Reni
Geonomateae	*Geonoma aspidiifolia*	Y	Reni, Con 3, Con 4
Geonomateae	*Geonoma deversa*	A	Con 4
Geonomateae	*Geonoma maxima*	Y, A	Reni, Con 3, Con 4
Geonomateae	*Geonoma stricta*	Y, A	Reni, Con 3, Con 4
Geonomateae	*Geonoma triglochin*	Y	Reni, Con 3, Con 4

Tribe, and if applicable subtribe, are shown (Dransfield et al., 2008). Hyperdominant species are shown in bold. Presence of species at each forest plot is indicated with Y for Yasuní, A for Amacayacu, and M for MPA. GE is Globular Echinate, GEE is Globular Elongate Echinate, GESP is Globular Echinate Short Projections, LGG is Large Globular Granulate, Reni Is Reniform, and Con is Conical. Unnumbered “Con” phytolith classification indicates that the species' phytolith production was categorized using older classification methods for conicals (i.e., identified only as a “conical” phytolith and not according to subsequently identified conical morphotypes). See Figure S4 for photos of the phytolith morphotypes.

We found 11 palm phytolith morphotypes in the samples analyzed from Yasuni, nine of which are known to be produced by palm species occurring in the plot (Table 2) and two unidentified types (star palm, “other” palm, see Figures S4–S5). The same 11 palm phytolith morphotypes identified at Yasuní were also identified at Amacayacu (Heijink et al., 2020), though at MPA palm phytoliths were quantified as spheroid palms (globular echinate), GESP (globular echinate short projections), or conical palms (Piperno et al., 2021). Conical type 4 phytoliths were only produced by *Geonoma* can be interpreted as such across all the locations (Table 2).

### 3.3 Phytolith assemblages Yasuní

We found a total of 26 different phytolith morphotypes in the soil cores collected at the Yasuní plot, including six arboreal morphotypes, 11 palm morphotypes, and eight grass morphotypes (Figure 3). Phytoliths indicating agricultural activities such as maize, rice, or manioc were not found in any sample. Most samples were dominated by arboreal phytoliths (33%–91%). The rugose arboreal morphotype ranged between 9% and 80%, and declined in all cores toward the core surface (Figure 3). Abundances of decorated arboreal spheres ranged between 4% and 59% and were overall less abundant than rugose spheres. In five out of ten cores, decorated sphere phytolith abundances were highest in the surface samples (Figure 3). Six out of ten cores showed an increase in decorated arboreal spheres around 20–40 cm and a subsequent decrease at 0–20 cm. This pattern was not detected in four cores with relatively high abundance of charcoal.

Abundances of globular echinate (GE) palm phytoliths at Yasuní, produced by *Attalea, Euterpe, Hyospathe, Mauritia, Oenocarpus*, and *Phytelephas*, ranged between 1% and 30%. Abundances slightly increased toward the upper depths in eight out of ten cores, although a decrease can be seen from 0 to 10 to the surface samples in nine out of ten cores (Figure 3). Globular echinate elongate (GEE) phytoliths (max 8%) had on average lower abundances than GE phytoliths and increased in 7 out of 10 cores excluding surface samples (Figure 3). Conical phytolith abundances varied between 3% and 56% and were on average higher than echinate abundances (Figure 3). Conical type 1, 2, and 3 phytoliths generally increased toward upper depths in the soil cores. A minimal increase was visible in conical type 4 (*Geonoma* sp.) in all cores. Total palm abundance increased toward the upper depths across almost all soil cores.

Grass phytoliths were found in 87% (78 out of 90) of the samples at Yasuní, and abundances ranged from 0% to 6% (Figure 3). *Heliconia* phytoliths, indicative of forest opening, occurred at less than 1% in five samples from five different cores. The individual grass morphotypes, total grass phytoliths, and *Heliconia* presence/abundance were not associated with charcoal abundances (Figure 3).

### 3.4 Fire and vegetation changes in northwestern Amazonia

Yasuní experienced more recent fires than Amacayacu or MPA (Figure 2a). The Kruskal–Wallis test indicated that charcoal abundances per core between the three sites were not significantly different (*p* = .11, chi‐squared = 4.37), but the proportions of samples within a core with charcoal differed significantly between Yasuní, Amacayacu, and MPA (*p* = <.0001, chi‐squared = 18.78) (Figure 2b and Figure S2). Pairwise Wilcoxon tests showed that MPA had significantly higher values than those at Yasuní and Amacayacu (*p* = <.001 for both MPA‐Ama and MPA‐Yasuní).

**FIGURE 4:**
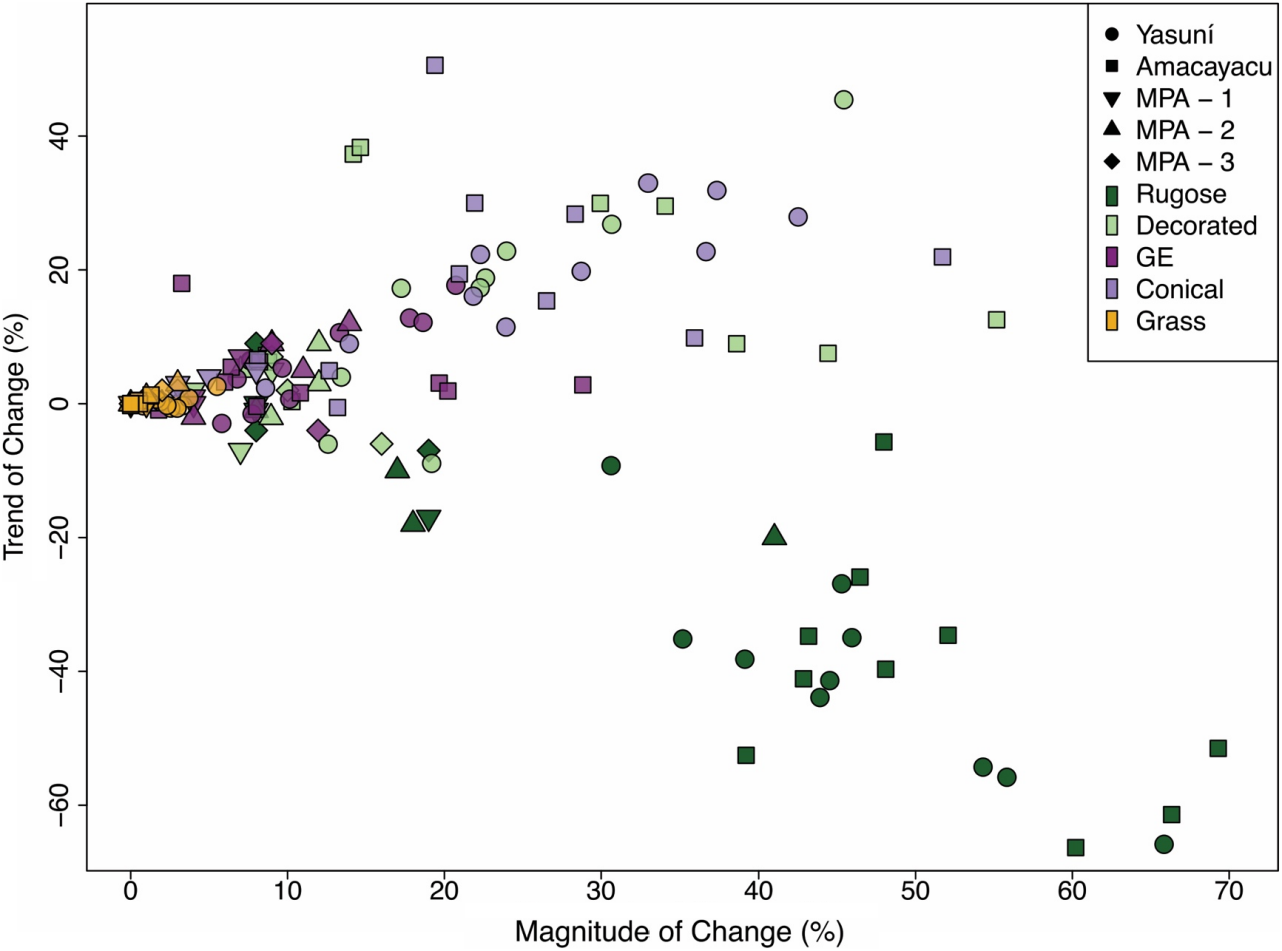
Magnitude of change plotted against trend of change for major arboreal, palm, and grass phytolith types. Major arboreal types include rugose spheres (darkgreen) and decorated spheres (lightgreen). Major palm types include globular echinate (darkpurple) and conical (lilac). Grasses are shown in yellow.

Yasuní and Amacayacu had higher variance in the trend and magnitude of change values for various phytolith types compared with the Medio Putumayo‐Algodón (MPA) site (Figure 4). The magnitude of change and trend of change values at Yasuní and Amacayacu had similar ranges in all categories expect for grass (Figures 3, 4), where Yasuní showed higher variability in the magnitude of change values. Yasuní showed a decrease in decorated spheres in cores 22 and 25 while decorated spheres increased in all cores in Amacayacu. All cores at Yasuní showed a positive trend of change in palms (both conical and echinate), whereas one core at Amacayacu showed a slight decrease.

**FIGURE 5:**
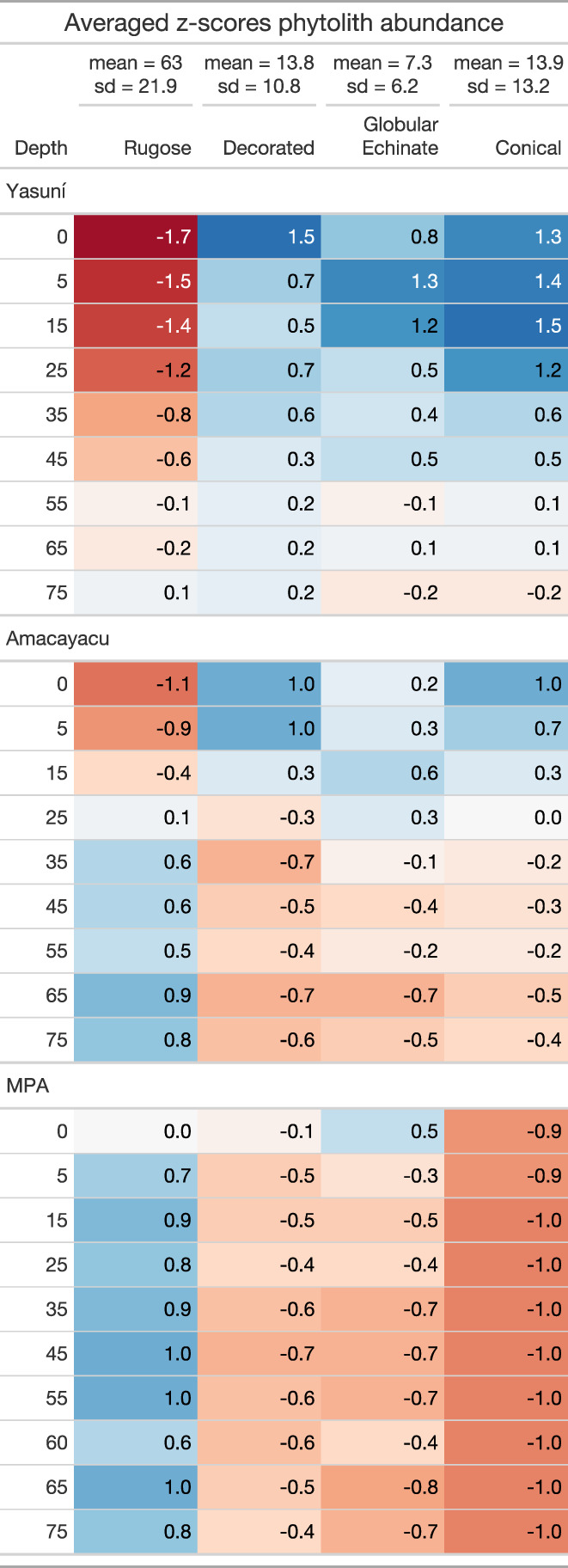
Z‐scores of phytolith types across all sites (Yasuní, Amacayacu, and MPA). Blue colors indicate a positive deviation from the regional mean (higher phytolith abundance), and red colors indicate a negative deviation from the regional mean (lower phytolith abundance).

The z‐scores for the major phytolith types show that vegetation patterns have fluctuated through time more at Yasuní and Amacayacu than at MPA (Figure 5). Trends in rugose sphere (arboreal) phytoliths oscillated within the Yasuní and Amacayacu cores, whereas at MPA they remained stable and at abundances higher than the regional average. The decorated sphere (arboreal) phytoliths also increased over time at Yasuní and Amacayacu, but at MPA abundances were stable and at abundances lower than the regional average (Figure 5). For the globular echinate and conical palm phytoliths, the z‐scores deviated to their highest point from the mean at depths <20 cm at Yasuní and Amacayacu, but remained well below the regional mean at MPA.

## 4 DISCUSSION

Fire is indicative of human activity in Amazonian rainforests, especially during the wetter conditions of the late Holocene (e.g., the last ca. 4000 years) (Bush et al., 2008; Malhi et al., 2008). The northwestern Amazonian forest plots analyzed here contain evidence of pre‐Columbian human occupation (fire presence), but have low numbers of datable charcoal fragments, low overall charcoal abundances, and no evidence of past cultivation, widespread fire, or large forest openings (Figures 2, 3). Though infrequent, forest burning was most common at the three sites between 2500 (550 BCE) and 1000 cal yr BP (950 CE) (Figure 2a), a period when archeological data show an increase in site use frequency and evidence of societies transitioning into a sedentary lifestyle across the basin (Neves et al., 2004; Piperno, 2011). The charcoal and phytolith data show that pre‐Columbian human occupation in the areas around the forest plots was intermittent and not widespread. The frequency and abundances of charcoal fragments and phytoliths indicating disturbance across our three study sites were lower than has been reported at other sites in central and eastern Amazonia where similar analyses have been performed on terrestrial soils (McMichael et al., 2012, 2015). Data from lake sediments have also indicated that the frequency of forest opening and burning by people in the pre‐Columbian era was lower in northwestern Amazonia compared with the southwestern and central regions of the basin (Gosling et al., 2021; Nascimento et al., 2022).

The Yasuní forest plot contains more frequent evidence of past small‐scale canopy openings, for example, the presence of *Heliconia* and increases of grass phytolith abundances, compared with MPA and Amacayacu (Figure 3 and Figures S6–S8). The trend of change and magnitude of change values, however, indicated that these forest openings were minor and did not persist through time (Figure 4). These small forest openings may have been the result of humans, natural disturbances, or both. For example, storms are known to uproot and blow‐down trees in the Yasuní region, creating canopy openings by downing dozens of trees in a single event. (Valencia, Foster, et al., 2004). Yasuní is the ancestral home of the Waorani people, who now use permanent settlements but in the past were highly mobile, semi‐nomadic hunters‐gatherers that used forest clearings for temporary settlements where they practiced horticulture (Beckerman et al., 2009; Finer et al., 2009; Lee et al., 1999). The Waorani may have exploited these natural disturbances, using fire intermittently.

The last recorded pre‐Columbian fire event at ca. 730 cal yr BP (1220 CE) at Yasuní corresponds with other records in northwestern Amazonia (Bush et al., 2021). Lakes Sauce and Kumpak^a^ (Figure 1) have documented decreases in the abundance and presence of maize microfossils at 750 (1200 CE) and 680 cal yr BP (1270 CE), respectively (Åkesson et al., 2021; Bush et al., 2016). Lake Quistococha, near the MPA region (Figure 1), also shows significant vegetation change around 680 cal yr BP (1270 CE) (Kelly et al., 2018). A reduction in human activity and corresponding forest regrowth from 950 to 1350 cal yr BP (650–1000 CE) also extends outside northwestern Amazonia, and have been documented in the central, eastern, and southwestern forests (Bush et al., 2021). The drivers behind the abandonment are unclear, but climatic instability associated with the Medieval Climate Anomaly (ca. 1000–700 cal yr BP, 950–1250 CE) and the Little Ice Age (400–150 cal yr BP, 1550–1800 CE) likely affected how pre‐Columbian people managed the land both in the Amazon and in the Andes (e.g., Åkesson et al., 2019; Ledru et al., 2013).

Significant increases in total palms and coincident decreases in total arboreal taxa were seen across all surveyed regions, and there was also an overturning of arboreal taxa during these transitions. In most cases, rugose sphere phytoliths were typically replaced by decorated (ornate and granulate) sphere phytoliths (Figures 3 and 4). Rugose spheres are known to be produced in high numbers by members of the Chrysobalanaceae family and in far lower abundances by a few other genera, including those with well‐distributed hyperdominant species such as *Eschweilera* (see Piperno & McMichael, 2020; Piperno et al., 2021 for taxa represented by rugose and other arboreal spheroids in the modern Amazonian flora and at MPA, respectively). The consistency of this replacement of rugose spheres or more decorated or ornate type suggested that this may have been a regional pattern of vegetation change occurring in the past.

The magnitude and trends of increases in palm abundances are stronger at Yasuní and Amacayacu than MPA, particularly with the conical phytolith types (Figures 4, 5). Conical phytoliths are produced by the hyperdominant species *Iriartea deltoidea* and *Socratea exhorriza* (Table 2). These two species are common at the Yasuní and Amacayacu plots in the modern landscape and occur in low abundances at MPA, which is consistent with the percentages of conical phytoliths in the surface samples of each site (Figures 3 and S7). Palms that produce globular echinate phytoliths, for example the hyperdominant species *Euterpe precatoria* and *Oenocarpus bataua*, appear to have also increased in abundance through the late‐Holocene at Yasuni and Amacayacu but more locally and sporadically than the taxa producing conical phytoliths (Figures 3, 4, Figures S6–S8).

We can place approximate ages on some of the vegetation changes that occurred at MPA (Piperno et al., 2021), but we do not have radiocarbon dates to infer the timing of these changes at Yasuní or Amacayacu. Soil cores collected similarly to ours in Amazonia typically capture the last several thousand years (McMichael et al., 2012;Piperno, 2016; Piperno et al., 2015, 2021). We can thus safely infer that the vegetation changes captured at all three sites occurred during this time window. We can also infer that the Yasuní and Amacayacu soils have retained some stratigraphic integrity because of the magnitude of change recorded throughout the cores at all three sites (Figures 3, 4, 5). If there were significant taphonomic problems or bioturbation problems with these soils cores, the phytolith assemblages would appear homogenous throughout, and the mixing would be evident.

Amazonian palm diversity is highest in the northwestern forests, where the distributions of eastern Amazonian and western Amazonian (and Andean) species coalesce and overlap (Montufar & Pintaud, 2006). Beta diversity of palms is also high in northwestern Amazonia; microtopography and local‐scale habitat heterogeneity are known to affect palm distributions and abundances (e.g., Balslev & Renner, 1989; Svenning, 1999a, 1999b, 2001). Disturbances, however, are also known to affect palm survival, mortality, and regeneration, and may also be playing a role in structuring modern patterns of palm occurrence and abundances (Montúfar et al., 2011). Palms are more resilient to blow‐down events compared with other tree taxa (Montúfar et al., 2011; Muscarella et al., 2020), and the repeated localized canopy openings at Yasuní (Figure 3), which occur with little to no fire, suggests that the frequency of natural disturbances or low levels of human impact could have contributed to an increase in palm abundances. As most palms have large seeds that are dispersed or predated by mammals (Beckman & Muller‐Landau, 2007; Markl et al., 2012; Wright et al., 2000; Wyatt & Silman, 2004), hunting may have also affected palm abundances in pre‐ and post‐Columbian times. The Waorani have lived around the Yasuní area for hundreds of years and lived as hunters‐and‐gatherers in pre‐modern times (Mena et al., 2000; Rival, 1999, 2016). Even though there is little evidence of past fire at Yasuní, indirect human legacies due to continuous hunting pressure may be driving the documented increases in certain palms.

Human activities during the last couple of hundred years may have left a stronger legacy on modern vegetation in the northwestern Amazonian forests than pre‐Columbian activities in some regions. The two surface samples from MPA showed relatively large changes in the phytolith abundances of arboreal taxa and palms compared with the sample collected from 0 to 10 cm (Figure 5). Phytoliths dated from a soil core at MPA‐2 indicate that the largest decreases in taxa producing rugose spheres and increases in taxa producing decorated sphere phytoliths occurred in the last four hundred years (Figure 5) (Piperno et al., 2021). The largest changes at Yasuní also occur in the most recent samples (e.g., surface samples compared with those found from 0 to 10 cm depth). Though we do not have phytolith dates from Yasuní, Amazonian surface samples typically date to the last several hundred years, whereas those found from 0 to 10 cm depth are typically older, often dating to the pre‐Columbian era (Piperno, 2016; Piperno et al., 2015, 2021). It is thus likely that more forest change occurred in last 400 years at Yasuní and MPA than during the pre‐Columbian era. The Rubber Boom in Amazonia occurred from 1850 to 1920 CE in regions where latex could be extracted from *Hevea brasiliensis* trees, which includes northwestern Amazonia (Hecht, 2013; Weinstein, 1983). The MPA region was known to be occupied during the Rubber Boom (Pitman et al., 2004), and even though people were not consistently using fire in the region, they were likely having a marked impact on the vegetation (Figure 5). The nearby Lake Quistococha (Figure 1) also shows heavier human disturbance in terms of deforestation during the last 200 years compared with those in the pre‐Columbian era (Kelly et al., 2018).

The onset of oil extraction is another major historical event in northwestern Amazonia (Finer et al., 2009) and likely also had an significant ecological influence. Beginning in the 1950 s, the northwestern portions of Amazonia, particularly in the Yasuní region, were surveyed for oil extraction (e.g., Bremner & Lu, 2006; Finer et al., 2009). The recent fire at 1956 CE within the Yasuní forest plot (Table S1) can potentially be explained by the presence of oil companies in Yasuní since 1940 and the subsequent conflicts between oil workers, Waorani, Taegeri, and Taromenane (Finer et al., 2009). It is also possible that a one‐time event such as a campfire was responsible for that dated charcoal fragment. The largest changes in vegetation composition at Yasuní also occur in the uppermost samples. Our data suggest that the observed ecological legacies do not only result from pre‐Columbian human activities, but also likely derive from much more recent disturbances during the last 200–400 years (Figure 5). Our data also suggest that patterns of hyperdominance change through time, and the configuration of modern hyperdominance results from both climatic and anthropogenic influences that varied from region‐to‐region during the late Holocene, particularly over the last few hundred years.

## AUTHOR CONTRIBUTIONS

B.M.H. and C.N.H.M. designed the study. C.N.H.M. collected samples in the field. A.P. processed all phytolith samples in the laboratorium. B.M.H. and Q.A.M. analyzed data and B.M.H. performed the statistical analyses. R.V. provided species data for Yasuni forest plot and was involved in fieldwork. B.M.H., D.R.P., and C.N.H.M. interpretated data. B.M.H. and C.N.H.M. led the writing of the manuscript with input and approval of all co‐authors.

## CONFLICT OF INTEREST

The corresponding author confirms on behalf of all authors that there have been no involvements that might raise the question of bias in the work reported or in the conclusions, implications, or opinions stated.

## Supporting information


supinfo

## Data Availability

The phytolith and charcoal data that support the findings of this study are openly available in the Dryad Digital Repository: (Heijink et al., 2022; https://doi.org/10.5061/dryad.tb2rbp042).

## References

[btp13175-bib-0001] Åkesson, C. M. , Matthews‐Bird, F. , Bitting, M. , Fennell, C.‐J. , Church, W. B. , Peterson, L. C. , Valencia, B. G. , & Bush, M. B. (2019). 2,100 years of human adaptation to climate change in the high Andes. Nature Ecology & Evolution, 4, 1–9. 10.1038/s41559-019-1056-2 31819239

[btp13175-bib-0002] Åkesson, C. M. , McMichael, C. N. , Raczka, M. F. , Huisman, S. N. , Palmeira, M. , Vogel, J. , Neill, D. , Veizaj, J. , & Bush, M. B. (2021). Long‐term ecological legacies in western Amazonia. Journal of Ecology, 109, 432–446.

[btp13175-bib-0003] Aleman, J. C. , Canal‐Subitani, S. , Favier, C. , & Bremond, L. (2014). Influence of the local environment on lacustrine sedimentary phytolith records. Palaeogeography, Palaeoclimatology, Palaeoecology, 414, 273–283.

[btp13175-bib-0004] Aleman, J. C. , Saint‐Jean, A. , Leys, B. , Carcaillet, C. , Favier, C. , & Bremond, L. (2013). Estimating phytolith influx in lake sediments. Quaternary Research, 80, 341–347.

[btp13175-bib-0005] Balée, W. (1988). Indigenous adaptation to Amazonian palm forests. Principes, 32, 47–54.

[btp13175-bib-0006] Balée, W. (1989). The culture of Amazonian forests. In D. A. Posey & W. Balée (Eds.), Resource management in Amazonia: Indigenous and folk strategies (pp. 1–21). New York Botanical Garden.

[btp13175-bib-0007] Balslev, H. , & Renner, S. (1989). Diversity of east Ecuadorean lowland forests. In L. B. Holm‐Nielsen , I. C. Nielsen , & H. Balslev (Eds.), Tropical Forests: Botanical Dynamics, Speciation and Diversity (pp. 287–295). Academic Press.

[btp13175-bib-0008] Barlow, J. , & Peres, C. A. (2008). Fire‐mediated dieback and compositional cascade in an Amazonian forest. Philosophical Transactions of the Royal Society B: Biological Sciences, 363, 1787–1794. 10.1098/rstb.2007.0013 PMC2373873 18267911

[btp13175-bib-0009] Bass, M. S. , Finer, M. , Jenkins, C. N. , Kreft, H. , Cisneros‐Heredia, D. F. , McCracken, S. F. , Pitman, N. C. A. , English, P. H. , Swing, K. , & Villa, G. (2010). Global conservation significance of Ecuador's Yasuní National Park. PLoS One, 5, e8767. 20098736 10.1371/journal.pone.0008767 PMC2808245

[btp13175-bib-0010] Beckerman, S. , Erickson, P. I. , Yost, J. , Regalado, J. , Jaramillo, L. , Sparks, C. , Iromenga, M. , & Long, K. (2009). Life histories, blood revenge, and reproductive success among the Waorani of Ecuador. Proceedings of the National Academy of Sciences, 106, 8134–8139. 10.1073/pnas.0901431106 PMC2688884 19433797

[btp13175-bib-0011] Beckman, N. G. , & Muller‐Landau, H. C. (2007). Differential effects of hunting on pre‐dispersal seed predation and primary and secondary seed removal of two Neotropical tree species. Biotropica, 39, 328–339.

[btp13175-bib-0012] Bernal, R. , Torres, C. , García, N. , Isaza, C. , Navarro, J. , Vallejo, M. I. , Galeano, G. , & Balslev, H. (2011). Palm Management in South America. The Botanical Review, 77, 607–646.

[btp13175-bib-0013] Blaauw, M. (2021). Package ‘IntCal’. R.

[btp13175-bib-0014] Bremner, J. , & Lu, F. (2006). Common property among indigenous peoples of the Ecuadorian Amazon. Conservation and Society, 4, 499–521.

[btp13175-bib-0015] Brienen, R. J. W. , Phillips, O. L. , Feldpausch, T. R. , Gloor, E. , Baker, T. R. , Lloyd, J. , Lopez‐Gonzalez, G. , Monteagudo‐Mendoza, A. , Malhi, Y. , Lewis, S. L. , Vasquez Martinez, R. , Alexiades, M. , Alvarez Davila, E. , Alvarez‐Loayza, P. , Andrade, A. , Aragao, L. E. O. C. , Araujo‐Murakami, A. , Arets, E. J. M. M. , Arroyo, L. , … Zagt, R. J. (2015). Long‐term decline of the Amazon carbon sink. Nature, 519, 344–348. 25788097 10.1038/nature14283

[btp13175-bib-0016] Bush, M. , Correa‐Metrio, A. , McMichael, C. , Sully, S. , Shadik, C. , Valencia, B. , Guilderson, T. , Steinitz‐Kannan, M. , & Overpeck, J. (2016). A 6900‐year history of landscape modification by humans in lowland Amazonia. Quaternary Science Reviews, 141, 52–64.

[btp13175-bib-0017] Bush, M. , Nascimento, M. , Åkesson, C. , Cárdenes‐Sandí, G. , Maezumi, S. , Behling, H. , Correa‐Metrio, A. , Church, W. , Huisman, S. , Kelly, T. , Mayle, F. , & McMichael, C. N. H. (2021). Widespread reforestation before European influence on Amazonia. Science, 372, 484–487. 33926948 10.1126/science.abf3870

[btp13175-bib-0018] Bush, M. B. , & McMichael, C. N. (2016). Holocene variability of an Amazonian hyperdominant. Journal of Ecology, 104, 1370–1378.

[btp13175-bib-0019] Bush, M. B. , Silman, M. R. , McMichael, C. , & Saatchi, S. (2008). Fire, climate change and biodiversity in Amazonia: A late‐Holocene perspective. Philosophical Transactions of the Royal Society B: Biological Sciences, 363, 1795–1802. 10.1098/rstb.2007.0014 PMC2373879 18267914

[btp13175-bib-0020] Chazdon, R. L. (2003). Tropical forest recovery: Legacies of human impact and natural disturbances. Perspectives in Plant Ecology, evolution and systematics, 6, 51–71.

[btp13175-bib-0021] Clement, C. R. (1988). Domestication of the pejibaye palm (Bactris gasipaes): Past and present. Advances in Economic Botany, 6, 155–174.

[btp13175-bib-0022] Clement, C. R. , de Cristo‐Araújo, M. , d'Eeckenbrugge, G. C. , Alves Pereira, A. , & Picanço‐Rodrigues, D. (2010). Origin and domestication of native Amazonian crops. Diversity, 2, 72–106.

[btp13175-bib-0023] Clement, C. R. , Denevan, W. M. , Heckenberger, M. J. , Junqueira, A. B. , Neves, E. G. , Teixeira, W. G. , & Woods, W. I. (2015). The domestication of Amazonia before European conquest. Proceedings of the Royal Society B, 282, 20150813. 26202998 10.1098/rspb.2015.0813 PMC4528512

[btp13175-bib-0024] Dickau, R. , Whitney, B. S. , Iriarte, J. , Mayle, F. E. , Soto, J. D. , Metcalfe, P. , Street‐Perrott, F. A. , Loader, N. J. , Ficken, K. J. , & Killeen, T. J. (2013). Differentiation of neotropical ecosystems by modern soil phytolith assemblages and its implications for palaeoenvironmental and archaeological reconstructions. Review of Palaeobotany and Palynology, 193, 15–37.

[btp13175-bib-0025] Dransfield, J. , Uhl, N. W. , Asmussen, C. B. , Baker, W. J. , Harley, M. M. , & Lewis, C. E. (2008). Genera Palmarum‐the evolution and classification of the palms.

[btp13175-bib-0026] Draper, F. C. , Costa, F. R. , Arellano, G. , Phillips, O. L. , Duque, A. , Macía, M. J. , Ter Steege, H. , Asner, G. P. , Berenguer, E. , & Schietti, J. (2021). Amazon tree dominance across forest strata. Nature ecology & evolution, 5, 757–767. 33795854 10.1038/s41559-021-01418-y

[btp13175-bib-0027] Fauset, S. , Johnson, M. O. , Gloor, M. , Baker, T. R. , Monteagudo, M. A. , Brienen, R. J. W. , Feldpausch, T. R. , Lopez‐Gonzalez, G. , Malhi, Y. , Ter Steege, H. , Pitman, N. C. A. , Baraloto, C. , Engel, J. , Pétronelli, P. , Andrade, A. , Camargo, J. L. C. , Laurance, S. G. W. , Laurance, W. F. , Chave, J. , … Phillips, O. L. (2015). Hyperdominance in Amazonian forest carbon cycling. Nature Communications, 6, 6857. 10.1038/ncomms7857 PMC4423203 25919449

[btp13175-bib-0028] Finer, M. , Vijay, V. , Ponce, F. , Jenkins, C. N. , & Kahn, T. R. (2009). Ecuador's Yasuni biosphere reserve: A brief modern history and conservation challenges. Environmental Research Letters, 4, 034005.

[btp13175-bib-0029] Franklin, J. F. , Lindenmayer, D. , MacMahon, J. A. , McKee, A. , Magnuson, J. , Perry, D. A. , Waide, R. , & Foster, D. (2000). Threads of continuity. Conservation Biology in Practice, 1, 8–16.

[btp13175-bib-0030] Gentry, A. H. (1988). Tree species richness of upper Amazonian forests. Proceedings of the National Academy of Sciences, 85, 156–159. 10.1073/pnas.85.1.156 PMC279502 16578826

[btp13175-bib-0031] Glaser, B. , & Woods, W. I. (2004). Amazonian dark earths: Explorations in space and time. Springer‐Verlag.

[btp13175-bib-0032] Gosling, W. D. , Maezumi, S. Y. , Heijink, B. M. , Nascimento, M. N. , Raczka, M. F. , van der Sande, M. T. , Bush, M. B. , & McMichael, C. N. (2021). Scarce fire activity in north and north‐western Amazonian forests during the last 10,000 years. Plant Ecology & Diversity, 14, 143–156.

[btp13175-bib-0033] Hartshorn, G. S. (1978). Tree falls and tropical forest dynamics. In P. B. Tomlinson & M. H. Zimmerman (Eds.), Tropical trees as living systems (pp. 617–638). Cambridge University Press.

[btp13175-bib-0034] Hecht, S. B. (2013). The scramble for the Amazon and the" lost paradise" of Euclides Da Cunha. University of Chicago Press.

[btp13175-bib-0035] Heijink, B. M. , Mattijs, Q. A. , Valencia, R. , Philip, A. L. , Piperno, D. R. , & McMichael, C. N. H. (2022). Data from: Long‐term fire and vegetation change in northwestern Amazonia. Dryad Digital Repository. doi:10.5061/dryad.tb2rbp042 PMC10108220 37081906

[btp13175-bib-0036] Heijink, B. M. , McMichael, C. N. , Piperno, D. R. , Duivenvoorden, J. F. , Cárdenas, D. , & Duque, Á. (2020). Holocene increases in palm abundances in North‐Western Amazonia. Journal of Biogeography, 47, 698–711.

[btp13175-bib-0037] Henderson, A. , Galeano‐Garces, G. , & Bernal, R. (1997). Field guide to the palms of the Americas. Princeton University Press.

[btp13175-bib-0038] Hijmans, R. J. , Cameron, S. E. , Parra, J. L. , & Jones, P. G. , & Jarvis, A. (2005). Very high resolution interpolated climate surfaces for global land areas. International Journal of Climatology, 25, 1965–1978.

[btp13175-bib-0040] Huisman, S. N. , Raczka, M. F. , & McMichael, C. N. H. (2018). Palm Phytoliths of mid‐elevation Andean forests. Frontiers in Ecology and Evolution, 6, 193.

[btp13175-bib-0041] John, R. , Dalling, J. W. , Harms, K. E. , Yavitt, J. B. , Stallard, R. F. , Mirabello, M. , Hubbell, S. P. , Valencia, R. , Navarrete, H. , & Vallejo, M. (2007). Soil nutrients influence spatial distributions of tropical tree species. Proceedings of the National Academy of Sciences, 104, 864–869. 10.1073/pnas.0604666104 PMC1783405 17215353

[btp13175-bib-0042] Kelly, T. J. , Lawson, I. T. , Roucoux, K. H. , Baker, T. R. , Honorio‐Coronado, E. N. , Jones, T. D. , & Rivas Panduro, S. (2018). Continuous human presence without extensive reductions in forest cover over the past 2500 years in an aseasonal Amazonian rainforest. Journal of Quaternary Science, 33, 369–379.

[btp13175-bib-0043] Ledru, M.‐P. , Jomelli, V. , Samaniego, P. , Vuille, M. , Hidalgo, S. , Herrera, M. , & Ceron, C. (2013). The medieval climate anomaly and the little ice age in the eastern Ecuadorian Andes. Climate of the Past, 9, 307–321.

[btp13175-bib-0044] Lee, R. B. , Daly, R. H. , & Daly, R. (1999). The Cambridge encyclopedia of hunters and gatherers. Cambridge University Press.

[btp13175-bib-0045] Lehman, J. , Kern, D. C. , Glaser, B. , & Woods, W. I. (2003). Amazonian dark earths: Origin, properties, management. Kluwer Academic Publisher, Dordrecht, The Netherlands.

[btp13175-bib-0046] Levis, C. , Costa, F. R. C. , Bongers, F. , Peña‐Claros, M. , Clement, C. R. , Junqueira, A. B. , Neves, E. G. , Tamanaha, E. K. , Figueiredo, F. O. G. , Salomão, R. P. , Castilho, C. V. , Magnusson, W. E. , Phillips, O. L. , Guevara, J. E. , Sabatier, D. , Molino, J.‐F. , López, D. C. , Mendoza, A. M. , Pitman, N. C. A. , et al. (2017). Persistent effects of pre‐Columbian plant domestication on Amazonian forest composition. Science, 355, 925–931. 28254935 10.1126/science.aal0157

[btp13175-bib-0047] Loughlin, N. J. , Gosling, W. D. , Mothes, P. , & Montoya, E. (2018). Ecological consequences of post‐Columbian indigenous depopulation in the Andean–Amazonian corridor. Nature Ecology & Evolution, 2, 1233–1236. 30013131 10.1038/s41559-018-0602-7

[btp13175-bib-0048] Macía, M. J. (2004). Multiplicity in palm uses by the Huaorani of Amazonian Ecuador. Botanical Journal of the Linnean Society, 144, 149–159.

[btp13175-bib-0049] Macía, M. J. , Armesilla, P. J. , Cámara‐Leret, R. , Paniagua‐Zambrana, N. , Villalba, S. , Balslev, H. , & Pardo‐de‐Santayana, M. (2011). Palm uses in northwestern South America: A quantitative review. The Botanical Review, 77, 462–570.

[btp13175-bib-0050] Malhi, Y. , Roberts, J. T. , Betts, R. A. , Killeen, T. J. , Li, W. , & Nobre, C. A. (2008). Climate change, deforestation, and the fate of the Amazon. Science, 319, 169–172. 18048654 10.1126/science.1146961

[btp13175-bib-0051] Markl, J. S. , Schleuning, M. , Forget, P. M. , Jordano, P. , Lambert, J. E. , Traveset, A. , Wright, S. J. , & Böhning‐Gaese, K. (2012). Meta‐analysis of the effects of human disturbance on seed dispersal by animals. Conservation Biology, 26, 1072–1081. 22971077 10.1111/j.1523-1739.2012.01927.x

[btp13175-bib-0052] McMichael, C. , Piperno, D. , Neves, E. , Bush, M. , Almeida, F. , Mongeló, G. , & Eyjolfsdottir, M. B. (2015). Phytolith assemblages along a gradient of ancient human disturbance in western Amazonia. Frontiers in Ecology and Evolution, 3, 141.

[btp13175-bib-0053] McMichael, C. , Piperno, D. R. , Bush, M. B. , Silman, M. R. , Zimmerman, A. R. , Raczka, M. F. , & Lobato, L. C. (2012). Sparse pre‐Columbian human habitation in western Amazonia. Science, 336, 1429–1431. 22700926 10.1126/science.1219982

[btp13175-bib-0054] McMichael, C. N. (2021). Ecological legacies of past human activities in Amazonian forests. New Phytologist, 229, 2492–2496. 32815167 10.1111/nph.16888 PMC7891632

[btp13175-bib-0055] Mena, V. , Stallings, J. R. , Regalado, J. , & Cueva, R. (2000). The sustainability of current hunting practices by the Huaorani. Hunting for Sustainability in Tropical Forests (pp. 57–78). Columbia University Press.

[btp13175-bib-0056] Montúfar, R. , Anthelme, F. , Pintaud, J.‐C. , & Balslev, H. (2011). Disturbance and resilience in tropical American palm populations and communities. The Botanical Review, 77, 426–461.

[btp13175-bib-0057] Montufar, R. , & Pintaud, J.‐C. (2006). Variation in species composition, abundance and microhabitat preferences among western Amazonian terra firme palm communities. Botanical Journal of the Linnean Society, 151, 127–140.

[btp13175-bib-0058] Morcote‐Ríos, G. , Bernal, R. , & Raz, L. (2016). Phytoliths as a tool for archaeobotanical, palaeobotanical and palaeoecological studies in Amazonian palms. Botanical Journal of the Linnean Society, 182, 348–360.

[btp13175-bib-0059] Morcote‐Rios, G. , Raz, L. , Giraldo‐Cañas, D. , Franky, C. E. , & León Sicard, T. (2013). Terras Pretas de Índio of the Caquetá‐Japurá River (Colombian Amazonia). Tipití: Journal of the Society for the Anthropology of Lowland South America, 11, 30–39.

[btp13175-bib-0060] Muscarella, R. , Emilio, T. , Phillips, O. L. , Lewis, S. L. , Slik, F. , Baker, W. J. , Couvreur, T. L. , Eiserhardt, W. L. , Svenning, J. C. , & Affum‐Baffoe, K. (2020). The global abundance of tree palms. Global Ecology and Biogeography, 29, 1495–1514.

[btp13175-bib-0061] Myers, N. , Mittermeier, R. A. , Mittermeier, C. G. , da Fonseca, G. A. B. , & Kent, J. (2000). Biodiversity hotspots for conservation priorities. Nature, 403, 853–858. 10706275 10.1038/35002501

[btp13175-bib-0062] Nascimento, M. N. , Heijink, B. M. , Bush, M. B. , Gosling, W. D. , & McMichael, C. N. H. (2022). Early to mid‐Holocene human activity exerted gradual influences on Amazonian forest vegetation. Philosophical Transactions of the Royal Society B: Biological Sciences, 377, 20200498. 10.1098/rstb.2020.0498 PMC8899618 35249380

[btp13175-bib-0063] Netherly, P. (1997). Loma y Ribera: patrones de asentamiento prehistóricos en la Amazonía Ecuatoriana. Fronteras de Investigación, 1, 33–54.

[btp13175-bib-0064] Neves, E. G. , Petersen, J. B. , Bartone, R. N. , & Heckenberger, M. J. (2004). The timing of *terra preta* formation in the Central Amazon: Archaeological data from three sites. In B. Glaser & W. I. Woods (Eds.), Amazonian dark earths: Explorations in space and time (pp. 125–133). Springer.

[btp13175-bib-0065] Pan, Y. , Birdsey, R. A. , Fang, J. , Houghton, R. , Kauppi, P. E. , Kurz, W. A. , Phillips, O. L. , Shvidenko, A. , Lewis, S. L. , & Canadell, J. G. (2011). A large and persistent carbon sink in the world's forests. Science, 333, 988–993. 21764754 10.1126/science.1201609

[btp13175-bib-0066] Parnell, A. (2016). Bchron: Radiocarbon dating, age‐depth modelling, relative sea level rate estimation, and non‐parametric phase modelling. R package version 4.1. 1; 2015.

[btp13175-bib-0067] Phillips, O. L. , Malhi, Y. , Higuchi, N. , Laurance, W. F. , Nunez, P. V. , Vasquez, R. M. , Laurance, S. G. , Ferreira, L. V. , Stern, M. , Brown, S. , & Grace, J. (1998). Changes in the carbon balance of tropical forests: Evidence from long‐term plots. Science, 282, 439–443. 9774263 10.1126/science.282.5388.439

[btp13175-bib-0068] Piperno, D. R. (2006). Phytoliths: A comprehensive guide for archaeologists and paleoecologists. Alta Mira Press.

[btp13175-bib-0069] Piperno, D. R. (2011). The origins of plant cultivation and domestication in the New World tropics. Current Anthropology, 52, S453–S470.

[btp13175-bib-0070] Piperno, D. R. (2016). Phytolith radiocarbon dating in archaeological and paleoecological research: A case study of phytoliths from modern Neotropical plants and a review of the previous dating evidence. Journal of Archaeological Science, 68, 54–61.

[btp13175-bib-0071] Piperno, D. R. , & Becker, P. (1996). Vegetational history of a site in the Central Amazon basin derived from phytolith and charcoal records from natural soils. Quaternary Research, 45, 202–209.

[btp13175-bib-0072] Piperno, D. R. , & McMichael, C. (2020). Phytoliths in modern plants from Amazonia and the neotropics at large: Implications for vegetation history reconstruction. Quaternary International, 565, 54–74.

[btp13175-bib-0073] Piperno, D. R. , McMichael, C. , & Bush, M. B. (2015). Amazonia and the Anthropocene: What was the spatial extent and intensity of human landscape modification in the Amazon Basin at the end of prehistory? The Holocene, 25, 1588–1597.

[btp13175-bib-0074] Piperno, D. R. , McMichael, C. H. , Pitman, N. C. , Andino, J. E. G. , Paredes, M. R. , Heijink, B. M. , & Torres‐Montenegro, L. A. (2021). A 5,000‐year vegetation and fire history for tierra firme forests in the Medio Putumayo‐Algodón watersheds, northeastern Peru. Proceedings of the National Academy of Sciences USA, 118, e2022213118. 10.1073/pnas.2022213118 PMC8501791 34580207

[btp13175-bib-0075] Piperno, D. R. , McMichael, C. N. , & Bush, M. B. (2019). Finding Forest Management in Prehistoric Amazonia. Anthropocene, 26, 100211.

[btp13175-bib-0076] Pitman, N. , Smith, R. C. , Vriesendorp, C. , Moskovits, D. K. , Piana, R. , Knell, G. , & Wachter, T. (2004). Perú: Ampiyacu, Apayacu, Yaguas. Field Museum, Environmental and Conservation Programs.

[btp13175-bib-0077] Pitman, N. C. , Terborgh, J. W. , Silman, M. R. , Núñez, P. , Neill, D. A. , Cerón, C. E. , Palacios, W. A. , & Aulestia, M. (2001). Dominance and distribution of tree species in upper Amazonian terra firme forests. Ecology, 82, 2101–2117.

[btp13175-bib-0078] Pitman, N. C. A. , Silman, M. R. , & Terborgh, J. W. (2013). Oligarchies in Amazonian tree communities: A ten‐year review. Ecography, 36, 114–123.

[btp13175-bib-0079] Poorter, L. , Bongers, F. , Aide, T. M. , Almeyda Zambrano, A. M. , Balvanera, P. , Becknell, J. M. , Boukili, V. , Brancalion, P. H. S. , Broadbent, E. N. , Chazdon, R. L. , Craven, D. , de Almeida‐Cortez, J. S. , Cabral, G. A. L. , de Jong, B. H. J. , Denslow, J. S. , Dent, D. H. , DeWalt, S. J. , Dupuy, J. M. , Durán, S. M. , … Rozendaal, D. M. A. (2016). Biomass resilience of Neotropical secondary forests. Nature, 530, 211–214. 26840632 10.1038/nature16512

[btp13175-bib-0080] Queenborough, S. A. , Burslem, D. F. , Garwood, N. C. , & Valencia, R. (2007). Habitat niche partitioning by 16 species of Myristicaceae in Amazonian Ecuador. Plant Ecology, 192, 193–207.

[btp13175-bib-0081] R Development Core Team . (2013). R: A language and environment for statistical computing. R Foundation for Statistical Computing.

[btp13175-bib-0082] Rasband, W. S. (2005). ImageJ 1.32j. National Institute of Health. USA.

[btp13175-bib-0083] Reimer, P. J. , Austin, W. E. , Bard, E. , Bayliss, A. , Blackwell, P. G. , Ramsey, C. B. , Butzin, M. , Cheng, H. , Edwards, R. L. , & Friedrich, M. (2020). The IntCal20 northern hemisphere radiocarbon age calibration curve (0–55 cal kBP). Radiocarbon, 62, 725–757.

[btp13175-bib-0084] Rival, L. (1999). The Huaorani. In Cambridge Encyclopedia of Hunters and Gatherers (pp. 101–104). Cambridge University Press.

[btp13175-bib-0085] Rival, L. (2016). Huaorani transformations in twenty‐first‐century Ecuador: Treks into the future of time. University of Arizona Press.

[btp13175-bib-0086] Roosevelt, A. C. (2013). The Amazon and the Anthropocene: 13,000 years of human influence in a tropical rainforest. Anthropocene, 4, 69–87.

[btp13175-bib-0087] Rozendaal, D. M. , Bongers, F. , Aide, T. M. , Alvarez‐Dávila, E. , Ascarrunz, N. , Balvanera, P. , Becknell, J. M. , Bentos, T. V. , Brancalion, P. H. , & Cabral, G. A. (2019). Biodiversity recovery of Neotropical secondary forests. Science Advances, 5, eaau3114. 30854424 10.1126/sciadv.aau3114 PMC6402850

[btp13175-bib-0088] Rudas Lleras, A. N. , Prieto Cruz, A. , Taylor, C. M. , & Ortiz, R. (2005). Flórula del Parque Nacional Natural Amacayacu. Universidad Nacional de Colombia, Facultad de Ciencias, Instituto de.

[btp13175-bib-0089] Rull, V. , & Montoya, E. (2014). Mauritia flexuosa palm swamp communities: Natural or human‐made? A palynological study of the gran Sabana region (northern South America) within a neotropical context. Quaternary Science Reviews, 99, 17–33.

[btp13175-bib-0090] Scott, A. C. (2010). Charcoal recognition, taphonomy and uses in palaeoenvironmental analysis. Palaeogeography, Palaeoclimatology, Palaeoecology, 291, 11–39.

[btp13175-bib-0091] Slik, J. F. , Arroyo‐Rodríguez, V. , Aiba, S.‐I. , Alvarez‐Loayza, P. , Alves, L. F. , Ashton, P. , Balvanera, P. , Bastian, M. L. , Bellingham, P. J. , & Van Den Berg, E. (2015). An estimate of the number of tropical tree species. Proceedings of the National Academy of Sciences, 112, 7472–7477. 10.1073/pnas.1423147112 PMC4475970 26034279

[btp13175-bib-0092] Svenning, J. C. (1999b). Microhabitat specialization in a species‐rich palm community in Amazonian Ecuador. Journal of Ecology, 87, 55–65.

[btp13175-bib-0093] Svenning, J.‐C. (1999a). Recruitment of tall arborescent palms in the Yasuni National Park, Amazonian Ecuador: Are large treefall gaps important? Journal of Tropical Ecology, 15, 355–366.

[btp13175-bib-0094] Svenning, J.‐C. (2001). On the role of microenvironmental heterogeneity in the ecology and diversification of neotropical rain‐forest palms (Arecaceae). The Botanical Review, 67, 1–53.

[btp13175-bib-0095] ter Steege, H. , Pitman, N. C. A. , Sabatier, D. , Baraloto, C. , Salomão, R. P. , Guevara, J. E. , Phillips, O. L. , Castilho, C. V. , Magnusson, W. E. , Molino, J.‐F. , Monteagudo, A. , Núñez Vargas, P. , Montero, J. C. , Feldpausch, T. R. , Coronado, E. N. H. , Killeen, T. J. , Mostacedo, B. , Vasquez, R. , Assis, R. L. , et al. (2013). Hyperdominance in the Amazonian tree Flora. Science, 342, 1243092. 24136971 10.1126/science.1243092

[btp13175-bib-0096] ter Steege, H. , Prado, P. I. , de Lima, R. A. , Pos, E. , de Souza Coelho, L. , de Andrade Lima Filho, D. , Salomão, R. P. , Amaral, I. L. , de Almeida Matos, F. D. , & Castilho, C. V. (2020). Biased‐corrected richness estimates for the Amazonian tree flora. Scientific Reports, 10, 1–13. 32576943 10.1038/s41598-020-66686-3 PMC7311553

[btp13175-bib-0097] Turner, M. G. (2010). Disturbance and landscape dynamics in a changing world. Ecology, 91, 2833–2849. 21058545 10.1890/10-0097.1

[btp13175-bib-0098] Uhl, C. , & Kauffman, J. B. (1990). Deforestation, fire susceptibility, and potential tree responses to fire in the eastern Amazon. Ecology, 71, 437–449.

[btp13175-bib-0099] Valencia, R. , Condit, R. , Foster, R. B. , Romoleroux, K. , Villa Munoz, G. , Svenning, J.‐C. , Magard, E. , Bass, M. , Losos, E. , & Balslev, H. (2004). Yasuni forest dynamics plot, Ecuador. Tropical Forest Diversity and Dynamism: Findings from a Large‐Scale Plot Network, 609, 620.

[btp13175-bib-0100] Valencia, R. , Foster, R. B. , Villa, G. , Condit, R. , Svenning, J. C. , Hernández, C. , Romoleroux, K. , Losos, E. , Magård, E. , & Balslev, H. (2004). Tree species distributions and local habitat variation in the Amazon: Large forest plot in eastern Ecuador. Journal of Ecology, 92, 214–229.

[btp13175-bib-0101] Watling, J. , Iriarte, J. , Whitney, B. , Consuelo, E. , Mayle, F. , Castro, W. , Schaan, D. , & Feldpausch, T. R. (2016). Differentiation of neotropical ecosystems by modern soil phytolith assemblages and its implications for palaeoenvironmental and archaeological reconstructions II: Southwestern Amazonian forests. Review of Palaeobotany and Palynology, 226, 30–43.

[btp13175-bib-0102] Weinstein, B. (1983). The Amazon rubber boom, 1850–1920. Stanford University Press.

[btp13175-bib-0103] Weng, C. (2005). An improved method for quantifying sedimentary charcoal via a volume proxy. The Holocene, 15, 298–301.

[btp13175-bib-0104] Witteveen, N. , Hobus, C. , Philip, A. , Piperno, D. , & McMichael, C. (2022). The variability of Amazonian palm phytoliths. Review of Palaeobotany and Palynology, 300, 104613.

[btp13175-bib-0105] Wright, S. J. , Zeballos, H. , Domínguez, I. , Gallardo, M. M. , Moreno, M. C. , & Ibáñez, R. (2000). Poachers alter mammal abundance, seed dispersal, and seed predation in a Neotropical forest. Conservation Biology, 14, 227–239.

[btp13175-bib-0106] Wyatt, J. L. , & Silman, M. R. (2004). Distance‐dependence in two Amazonian palms: Effects of spatial and temporal variation in seed predator communities. Oecologia, 140, 26–35. 15085424 10.1007/s00442-004-1554-y

