# Supplementary material for: Long‐term fire and vegetation change in northwestern Amazonia
Source: Biotropica. 2022 Dec 2;55(1):197–209. doi: 10.1111/btp.13175 (PMC10108220; doi:10.1111/btp.13175)
Supplement: Supplementary file 1 — supinfo [file BTP-55-197-s001.docx]

SUPPORTING INFORMATION

**Long-term fire and vegetation change in northwestern Amazonia**

Britte M. Heijink - Department of Ecosystem and Landscape Dynamics, Institute for Biodiversity and Ecosystem Dynamics, University of Amsterdam, the Netherlands; ORCID: 0000-0001-7359-5023

Quinten A. Mattijs - Department of Ecosystem and Landscape Dynamics, Institute for Biodiversity and Ecosystem Dynamics, University of Amsterdam, the Netherlands; ORCID: 0000-0002-1547-8985

Annemarie Philip - Department of Ecosystem and Landscape Dynamics, Institute for Biodiversity and Ecosystem Dynamics, University of Amsterdam, the Netherlands;

Renato Valencia - Escuela de Ciencias Biológicas, Pontificia Universidad Católica del Ecuador, Quito, Ecuador; ORDCID:0000-0001-9770-6568

Dolores R. Piperno - Department of Anthropology, Smithsonian National Museum of Natural History, Washington, DC, USA; Smithsonian Tropical Research Institute, Balboa, Panama; ORCID: 0000-0002-0584-3133

Crystal N.H. McMichael - Department of Ecosystem and Landscape Dynamics, Institute for Biodiversity and Ecosystem Dynamics, University of Amsterdam, the Netherlands; ORCID: 0000-0002-1064-1499

**Appendix S1.**

*Palm-phytolith relationships*

*Attalea, Euterpe, Hyospathe, Mauritia, Oenocarpus,* and *Phytelephas* all produced globular echinate (GE) and globular echinate elongate (GEE) phytoliths except for *Phytelephas* and *Mauritia* which only produced GE phytoliths (Table 2). *Hyospathe* and *Prestoea* produced reniform phytoliths, and globular echinate short spine phytoliths (GESP; also known as SAP, see Witteveen et al., 2022 were produced by *Prestoea*, *Euterpe*, and *Oenocarpus.* Large globular granulate (LGG) types were only produced by *Euterpe.* The four conical phytolith types were produced by *Aiphanes, Astrocaryum, Bactris, Chamaedora, Geonoma, Iriartea, Socratea,* and *Wettinia* (Table 2)*.* Conical type 4 phytoliths were only produced by *Geonoma*. *Iriartea* and *Socratea* only produced conical type 1 phytoliths (Table 2). At Amacayacu, the same 11 palm phytolith morphotypes were also analyzed (Heijink et al., 2020), though at MPA palm phytoliths were quantified as either spheroid palms (globular echinate), GESP, or conical (Piperno et al. 2021$).

*Modern 14C date Yasuní*

The charcoal fragment from Yasuní core Y2 was dated as ‘modern’ (Table S1). Calibration of this date gave two possibilities for the actual age, either 1956 CE or 2012 CE (Fig. S2). We interpreted this calibration as 1956 CE, even though 2012 CE has higher probability according to the calibration curve. However, the Yasuní forest plot was established in 1995 and heaviliy studied year-round. No fire has been reported in that time period, so we conclude that the charcoal fragment was burned around 1956 CE.

**Supplemental tables**

**Table S1. Radiocarbon dates (^14^C) of Yasuní, Amacayacu, and MPA.**  Calibrated ages correspond with the minimal, median, and maximum calibrated age in years Before Present in a 95 % confidence interval. Calibrations were performed using the IntCal20 calibration curve (Reimer et al., 2020). See Fig S1 for the calibration curve of the modern date from Yasuní core Y2. Radiocarbon dates from Amacayacu were previously published in Heijink et al (2020) and radiocarbon dates from MPA by Piperno et al (2022).

| **Site** | **Core** | **Depth** | **Uncalibrated date** | **Minimum Calibrated Age BP** | **Median calibrated Age BP** | **Maximum Calibrated Age BP** |
| --- | --- | --- | --- | --- | --- | --- |
| Yasuní | Y2 | 0-10 | Modern (pMC 103.07 +- 0.29) |  | 1956 CE |  |
| Yasuní | Y5 | 30-40 | 821 ± 25 | 683 | 715 | 772 |
| Yasuní | Y25 | 10-20 | 857 ± 21 | 704 | 754 | 788 |
| Amacayacu | AMA 6 | 0-10 | 1729 ± 32 | 1545 | 1618 | 1700 |
| Amacayacu | AMA 6 | 10-20 | 1833 ± 24 | 1643 | 1732 | 1817 |
| Amacayacu | AMA 6 | 20-30 | 1773 ± 27 | 1591 | 1651 | 1721 |
| Amacayacu | AMA 6 | 30-40 | 1825 ± 27 | 1636 | 1726 | 1817 |
| Amacayacu | AMA 10 | 40-50 | 1925 ± 28 | 1746 | 1840 | 1922 |
| Amacayacu | AMA 15 | 50-60 | 2467 ± 27 | 2375 | 2590 | 2704 |
| Amacayacu | AMA 15 | 50-60 | 2470 ± 27 | 2381 | 2573 | 2702 |
| MPA-1 | MPA-1 Core 1 | 30-40 | 1659 ± 26 | 1425 | 1543 | 1682 |
| MPA-1 | MPA-1 Core 3 | 0-10 | 2121 ± 29 | 2004 | 2086 | 2284 |
| MPA-1 | MPA-2 Core 1 | 20-30 | 2675 ± 43 | 2741 | 2785 | 2868 |
| MPA-2 | MPA-2 Core 2 | 40-50 | 2423 ± 30 | 2360 | 2449 | 2684 |
| MPA-2 | MPA-2 Core 3 | 60-70 | 2245 ± 28 | 2157 | 2225 | 2333 |
| MPA-3 | MPA-3 Core 4 | 30-40 | 1962 ± 27 | 1809 | 1890 | 1979 |

**Table S2. Charcoal metrics per site**

|  | **Yasuní** | **Amacayacu** | **MPA - all** | **MPA-1** | **MPA-2** | **MPA-3** |
| --- | --- | --- | --- | --- | --- | --- |
| Mean charcoal abundance (mm^3^/cm^3^) | 0.38 | 0.33 | 0.37 | 0.54 | 0.44 | 0.06 |
| Maximum charcoal abundance (mm^3^/cm^3^) | 15.37 | 18.60 | 6.57 | 6.57 | 3.29 | 0.21 |
| Proportion of samples containing charcoal | 0.25 | 0.25 | 0.82 | 0.82 | 0.94 | 0.65 |
| Proportion of cores containing charcoal | 0.76 | 0.67 | 1 | 1 | 1 | 1 |


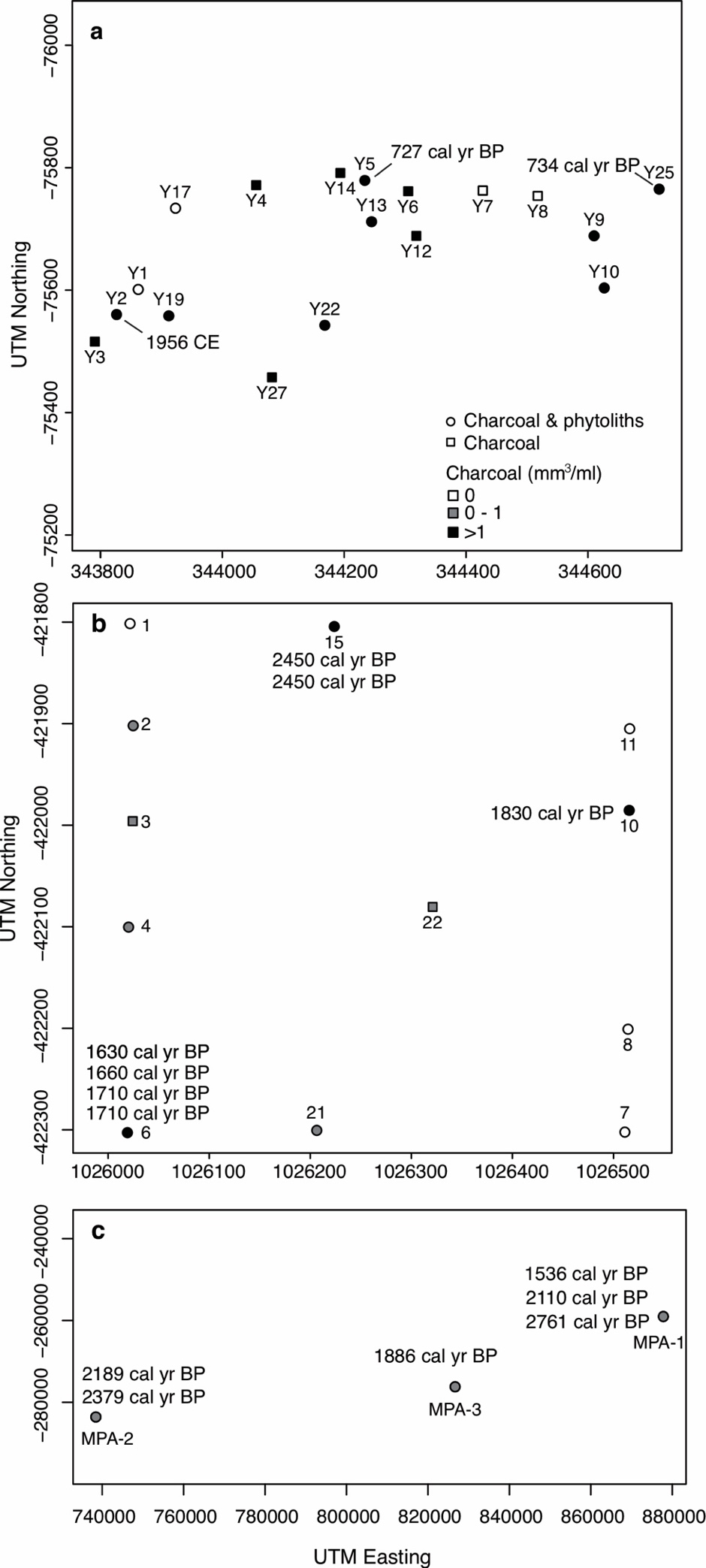
**Supplemental figures**

**Fig S1.** Close-ups of the 50-ha Yasuní forest plot (a) and the 25-ha Amacayacu forest plot (b) showing individual soil core locations and spatial configuration on the ^14^C AMS dated charcoal fragments. Panel c shows subplot locations MPA-1, MPA-2, and MPA-3. Within these subplots cores were taken along a vegetation transect (Piperno et al., 2021). Charcoal abundances are color-coded. Circles indicate sites that were analyzed for both charcoal and phytoliths, squares indicate sites that were analyzed for charcoal. Calibrated ages are in calibrated years Before Present.

**Fig S2. Calibration curve of the modern date at Yasuní core Y2.** X-axis represents the calibrated age Before Present, y-axis the uncalibrated age.


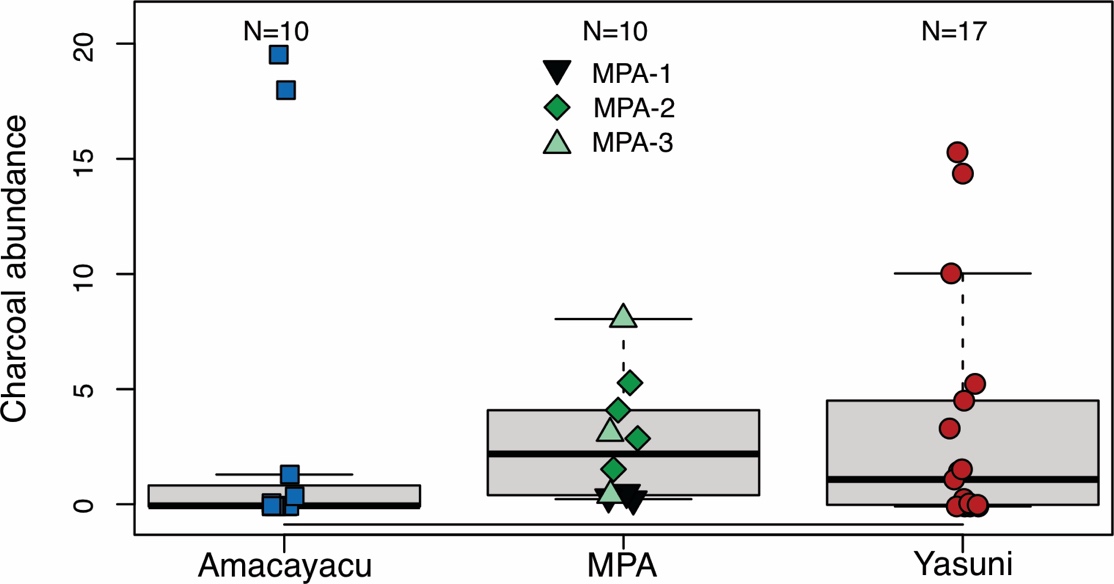


**Fig S3.** Boxplot of charcoal abundances per sample in mm^3^/cm^3^ across Amacayacu (blue), MPA, (green), and Yasuní (red). MPA-1 is indicated with upside down triangles (darkgreen), MPA-2 with diamonds (green), and MPA-3 with triangles (lightgreen)


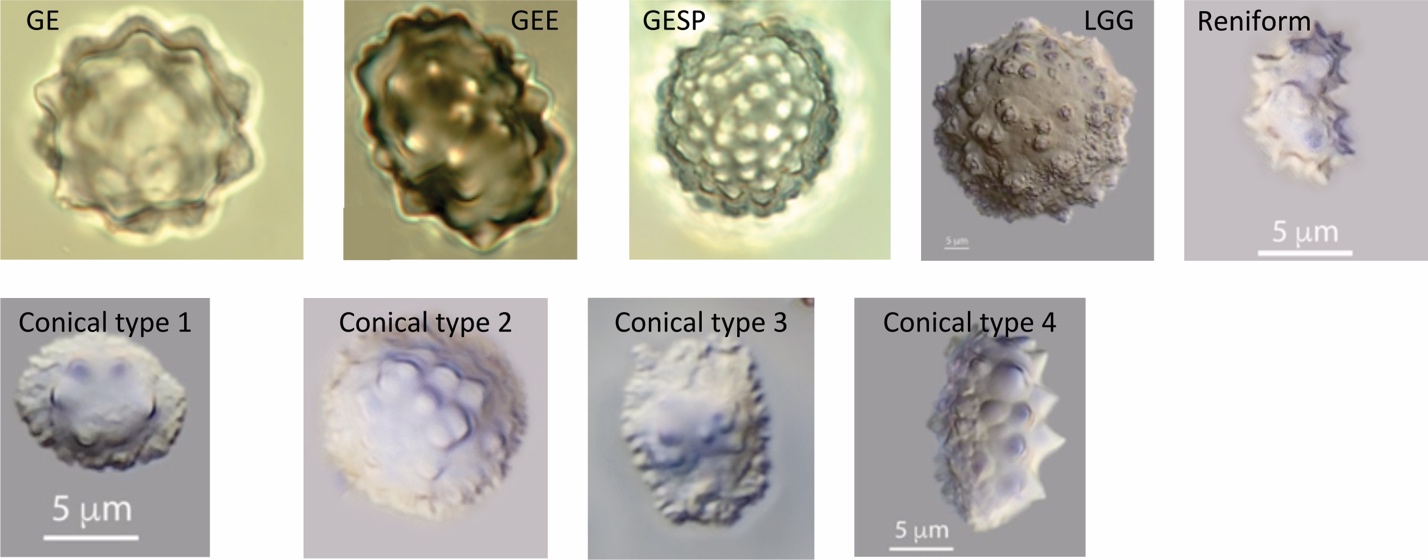


**Fig. S4: Palm phytolith morphotypes as described in Table 2.**


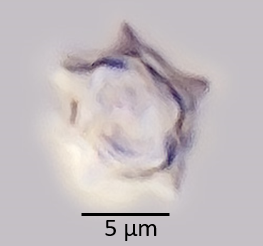


**Fig. S5: Photo of ‘star palm’ phytolith with unknown origin.**

**Fig S6.** Stratigraphic diagram of phytolith and charcoal abundances found in soil cores in the Amacayacu forest plot. Echinate shows the sum of all echinate phytolith types, including GE, GEE, GESP, reniform, and star palm. Conical shows the sum of all conical phytolith abundances. Charcoal abundances are in mm^3^/cm^3^.

**Fig. S7.** Stratigraphic diagram of phytolith and charcoal abundances found in soil cores in the MPA plots. Echinate shows the sum of all echinate phytolith types. Conical shows the sum of all conical phytoliths abundances. Charcoal abundances are in mm^3^/cm^3^.

**Fig S8.** Stratigraphic diagram of percentages of all phytoliths types occurring in the Yasuní forest plot. Charcoal abundances are in mm^3^/cm^3^.

**References**

Heijink, B. M., McMichael, C. N., Piperno, D. R., Duivenvoorden, J. F., Cárdenas, D. & Duque, Á. (2020) Holocene increases in palm abundances in north‐western Amazonia. *Journal of Biogeography,* **47,** 698-711.

Piperno, D. R., McMichael, C. H., Pitman, N. C., Andino, J. E. G., Paredes, M. R., Heijink, B. M. & Torres-Montenegro, L. A. (2021) A 5,000-year vegetation and fire history for tierra firme forests in the Medio Putumayo-Algodón watersheds, northeastern Peru. *Proceedings of the National Academy of Sciences*.

Reimer, P. J., Austin, W. E., Bard, E., Bayliss, A., Blackwell, P. G., Ramsey, C. B., Butzin, M., Cheng, H., Edwards, R. L. & Friedrich, M. (2020) The IntCal20 Northern Hemisphere radiocarbon age calibration curve (0–55 cal kBP). *Radiocarbon,* **62,** 725-757.

Witteveen, N., Hobus, C., Philip, A., Piperno, D. & McMichael, C. (2022) The variability of Amazonian palm phytoliths. *Review of Palaeobotany and Palynology,* **300,** 104613
